# Toxoplasma gondii Intravacuolar-Network-Associated Dense Granule Proteins Regulate Maturation of the Cyst Matrix and Cyst Wall

**DOI:** 10.1128/mSphere.00487-19

**Published:** 2019-10-16

**Authors:** Rebekah B. Guevara, Barbara A. Fox, Alejandra Falla, David J. Bzik

**Affiliations:** a Department of Microbiology and Immunology, The Geisel School of Medicine at Dartmouth, Lebanon, New Hampshire, USA; University of Georgia

**Keywords:** *Toxoplasma gondii*, cysts, chronic infection, cyst wall, cyst matrix, intravacuolar network, dense granule proteins, bradyzoite differentiation, cyst development, cyst maturation

## Abstract

Little is known regarding how the chronic Toxoplasma gondii cyst develops. Here, we investigated intravacuolar-network-associated dense granule (GRA) proteins GRA1, GRA2, GRA4, GRA6, GRA9, and GRA12 during cyst development *in vitro* after differentiation of the tachyzoite-stage parasitophorous vacuole. By day 1 postdifferentiation, GRA1, GRA4, GRA6, GRA9, and GRA12 colocalized with Dolichos biflorus agglutinin stain at the cyst periphery. In contrast, GRA2 remained in the cyst matrix. By day 2 postdifferentiation, coinciding with localization of GRA2 to the cyst periphery, GRA1, GRA4, GRA6, and GRA9 established a continuous matrix pattern in the cyst. In contrast, GRA2 and GRA12 were colocalized in prominent cyst matrix puncta throughout cyst development. While GRA2, GRA6, and GRA12 localized in outer and inner layers of the cyst wall, GRA1, GRA4, and GRA9 localized predominantly in the inner layers of the cyst wall. GRA2 and GRA12 were colocalized in the cyst wall by day 7 postdifferentiation. However, by day 10 postdifferentiation, GRA12 was relocalized from the cyst wall to puncta in the cyst matrix. Differentiation of Δ*gra2* parasites revealed a defect in the ability to establish a normal cyst matrix. In addition, the deletion of any intravacuolar-network-associated GRA protein, except GRA1, reduced the rate of accumulation of cyst wall proteins at the cyst periphery relative to the cyst interior. Our findings reveal dynamic patterns of GRA protein localization during cyst development and suggest that intravacuolar-network-associated GRA proteins regulate the formation and maturation of the cyst matrix and cyst wall structures.

**IMPORTANCE** Toxoplasma gondii establishes chronic infection in humans by forming thick-walled cysts that persist in the brain. If host immunity wanes, cysts reactivate to cause severe, and often lethal, toxoplasmic encephalitis. There is no available therapy to eliminate cysts or to prevent their reactivation. Moreover, how the vital and characteristic cyst matrix and cyst wall structures develop is poorly understood. Here, we visualized and tracked the localization of *Toxoplasma* intravacuolar-network-associated dense granule (GRA) proteins during cyst development *in vitro*. Intravacuolar-network GRAs were present within the cyst matrix and at the cyst wall in developing cysts, and genetic deletion of intravacuolar-network-associated GRAs reduced the rate of accumulation of cyst wall material at the cyst periphery. Our results show that intravacuolar-network-associated GRAs, particularly GRA2 and GRA12, play dynamic and essential roles in the development and maturation of the cyst matrix and the cyst wall structures.

## Abstract (precis)

Toxoplasma gondii establishes chronic infection in humans by forming thick-walled cysts that persist in the brain. If host immunity wanes, cysts reactivate to cause severe, and often lethal, toxoplasmic encephalitis. There is no available therapy to eliminate cysts or to prevent their reactivation. Moreover, how the vital and characteristic cyst matrix and cyst wall structures develop is poorly understood. Here, we visualized and tracked the localization of *Toxoplasma* intravacuolar-network-associated dense granule (GRA) proteins during cyst development *in vitro*. Intravacuolar-network GRAs were present within the cyst matrix and at the cyst wall in developing cysts, and genetic deletion of intravacuolar-network-associated GRAs reduced the rate of accumulation of cyst wall material at the cyst periphery. Our results show that intravacuolar-network-associated GRAs, particularly GRA2 and GRA12, play dynamic and essential roles in the development and maturation of the cyst matrix and the cyst wall structures.

## INTRODUCTION

A third of the human population is chronically infected by Toxoplasma gondii (1). *Toxoplasma* converts into four different cellular stages: sporozoite, merozoite, tachyzoite, and bradyzoite (2). Natural transmission of *Toxoplasma* is achieved through walled structures that contain transmissible parasite stages, sporozoites are inside oocysts, and bradyzoites are inside tissue cysts (2). *Toxoplasma* infection occurs after ingestion of oocysts in water or on unwashed fruits and vegetables or via ingestion of tissue cysts in undercooked meat (3). Infection of the fetus during pregnancy may cause stillbirth or miscarriage (4), and newborns often present with significant congenital manifestations (5, 6). If infection occurs in the immune-privileged eye, severe inflammation of the retina leads to recurrent ocular toxoplasmosis. During immune deficiency, reactivation of tissue cysts can cause life-threatening toxoplasmic encephalitis (7–9). The biology underlying the development of tissue cysts is poorly understood, and current therapies do not target the cyst stage.

Acute *Toxoplasma* infection is characterized by tachyzoite-stage parasites that actively invade host cells by motility-driven invagination of the host plasma membrane to form a vacuole, called the parasitophorous vacuole (PV) (10). Inside the PV, tachyzoites replicate until their egress and infection of new host cells (11, 12). If infection occurs near the blood-brain barrier, tachyzoite-stage parasites can breach this barrier and subsequently invade neurons (13, 14), an important host cell for the parasite’s differentiation from the tachyzoite stage to the encysted bradyzoite stage (15, 16). Bradyzoite-stage parasites phenotypically divide slowly and are encased within a cyst matrix surrounded by a thick cyst wall (17, 18). The cyst matrix occupies the spaces between the bradyzoites and the cyst wall. Soluble components, filamentous materials, membranous tubules, and vesicles reside in the cyst matrix (19). The cyst wall has a granular appearance and a thickness of ∼270 to 850 nm (20) and is organized into two distinct filamentous layers, a more densely compacted outer layer beneath a limiting cyst membrane and a less densely compacted inner layer that faces the cyst matrix (19). The cyst wall structure of *in vitro*-derived cysts is highly similar to the cyst wall structure of *in vivo* cysts that have developed in the brains of infected mice (21). An established *in vitro* tachyzoite-to-bradyzoite differentiation method that exposes the tachyzoite-stage PV to low CO_2_ and a medium with a higher pH (22, 23) triggers cyst development *in vitro* and produces mature orally infectious cysts from all cyst-competent type I, type II, type III, and exotic strain types (24). These *in vitro* cysts become orally infectious after 5 days of *in vitro* development, and cyst oral infectivity increases as cysts mature to 9 days of age (24).

The cyst wall contains several prominent glycoproteins, including the major cyst wall glycoprotein CST1. CST1 is the selective binding target of the cyst wall-specific Dolichos biflorus agglutinin (DBA) stain (17). The cyst wall contains another, unidentified glycoprotein that selectively binds to succinylated wheat germ agglutinin (25–27). DBA binds *N*-acetylgalactosamine moieties (25) that heavily decorate a mucin domain in CST1 (17, 28). CST1 localizes throughout granular material in the cyst wall, and genetic deletion of CST1 results in the loss of this granular material, thin cyst walls, and fragile cysts (17). A recent proteomic study of the cyst wall revealed that certain dense granule (GRA) proteins were localized in the mature cyst wall 8 days after differentiation of tachyzoite-stage PVs *in vitro* (29).

Tachyzoites massively secrete the dense granules and their GRA proteins into the PV space soon after the PV initially forms. These GRAs modify the nascent PV and the host cell environment (30, 31). Shortly after GRA protein secretion, the intravacuolar network (IVN) forms inside the PV to establish a network of highly curved membrane tubules (32, 33). The IVN membrane tubules connect parasites to one another and to the limiting PV membrane (PVM), and these tubules contain F-actin filaments that are 5 nm thick, that are more than 100 nm long, and that appear to be important in organizing the IVN, in forming the residual body, and in moving vesicles between tachyzoites in the PV (34–36). The IVN membranes also play an important role in lipid salvage from the host (37, 38) and acquisition of host cytosolic proteins (39). GRA1 peripherally associates with the IVN (40). In contrast, GRA2 (40), GRA4 (41), GRA6 (42), GRA9 (43), and GRA12 (44) strongly associate with the IVN membranes. In addition, the highly curved nanotubular architecture of the IVN is shaped by GRA2 (32, 45, 46) and GRA6 (32, 46). GRA2 induces the formation of curved tubules from vesicular material secreted from the posterior end of the parasite, and GRA6 stabilizes the curvature of these membrane tubules (32). However, unlike GRA2 and GRA6, GRA12 is not required for the formation of the IVN membrane tubules (47). Deletion of GRA2 led to a reduction of host cytosolic proteins ingested, which indicates that the IVN membrane tubules contribute to ingestion of host cytosol (39). The presence of small pores in the PVM has been shown using fluorescent dyes (48). Furthermore, GRA17 and GRA23 facilitate the movement of small molecules across the PVM (49). The roles of the IVN membranes and the GRA proteins that associate with the IVN during the development of the cyst after differentiation of the tachyzoite-stage PV are unknown.

Deletion of IVN-associated GRA2, GRA4, GRA6, GRA9, or GRA12 caused a major defect in the development of cyst burdens during chronic infection (47, 50). These recent findings suggested that IVN-associated GRA proteins could play important roles in the development of cysts. It has been suggested that the IVN membranes could be actively involved in cyst development after tachyzoite-to-bradyzoite stage differentiation is triggered (19, 51). The IVN membranes structurally resemble similar curved membrane tubules present in cysts, the intracyst network (ICN), that connects bradyzoites to each other in the cyst matrix and to the cyst wall (18, 19).

Here, we determined the locations of IVN-associated GRA proteins during cyst development. Our findings show that deletion of IVN-associated GRAs significantly reduced the rate of accumulation of cyst wall proteins at the cyst periphery relative to the cyst interior, suggesting that these IVN GRA proteins play an important role in the development of the cyst wall. GRA2 expression was essential for normal development of the cyst matrix and accumulation of GRA4 and GRA6 at the cyst periphery. IVN-associated GRA2 and GRA12 showed dynamic patterns of relocalization between the cyst matrix and the cyst wall. Collectively, our results suggest that IVN-associated GRAs play vital roles in the formation and maturation of the cyst matrix and cyst wall structures.

## RESULTS

### IVN-associated GRA mutants.

IVN-associated GRA deletion mutants that do not express GRA2, GRA4, GRA6, GRA9, or GRA12 were previously developed in the low-virulence cyst-competent type II Prugniaud (Pru) Δ*ku80* genetic background (47, 50). Here, we targeted the deletion of GRA1 (TGME49_270250) (see Fig. S1A in the supplemental material). The Δ*gra1* mutant was obtained, and the absence of GRA1 expression was confirmed by PCR validation and immunofluorescence assay (Fig. S1B). Since the gene encoding the prominent cyst matrix antigen MAG1 (TGME49_270240) resides immediately adjacent to GRA1, where GRA1 and MAG1 share an upstream noncoding regulatory region (Fig. S1A) (52), we verified that deletion of GRA1 did not affect the expression of MAG1 (Fig. S1C).

10.1128/mSphere.00487-19.1 FIG S1 GRA1 knockout and GRA12 complementation strategy. (A) Knockout strategy to insert *HXGPRT* at the deleted *GRA1* gene locus by selection with mycophenolic acid (MPA) and xanthine (X). As indicated in the schematic, the GRA1 gene and the MAG1 gene are neighbors on the chromosome. Double crossovers occurred at the *GRA* gene of interest (GOI) locus when the PruΔ*ku80*Δ*hxgprt* strain allowed the replacement of the targeted *GRA* coding sequence by that of the *HXGPRT* resistance marker, leading to the isolation of the PruΔ*ku80*Δ*gra1::HXGPRT* strain. The locations of the genotype validation PCRs are shown above the schematized PruΔ*ku80*Δ*gra1::HXGPRT* genome. The genotype of the GRA1 knockout was verified by PCR (not shown). (B and C) The PruΔ*gra1* strain lacks expression of GRA1 and retains expression of MAG1. Host HFF cells on coverslips were infected with an MOI of 0.5 tachyzoite of each strain, PruΔ*ku80* or PruΔ*ku80*Δ*gra1* (Δ*gra1*), permeabilized with 0.01% saponin, and stained with α-GRA1 (B) or α-MAG1 (C) (shown in red). DAPI (shown in blue) stained the host cell and parasite nuclei. PVs were located using DIC microscopy, and representative images are shown. Scale bars = 5 μm. (D) Complementation strategy at the original locus through selection with 6TX (6-thioxanthine). Design of genotype validation PCR is shown. The genotype of the GRA12 complementation was verified by PCR (not shown). Download FIG S1, TIF file, 1.2 MB. Copyright © 2019 Guevara et al. 2019 Guevara et al. This content is distributed under the terms of the Creative Commons Attribution 4.0 International license.

### DBA staining intensity at the cyst periphery relative to the cyst interior is decreased in mutant strains that lack expression of IVN-associated GRA proteins, except for GRA1.

**FIG 1:**
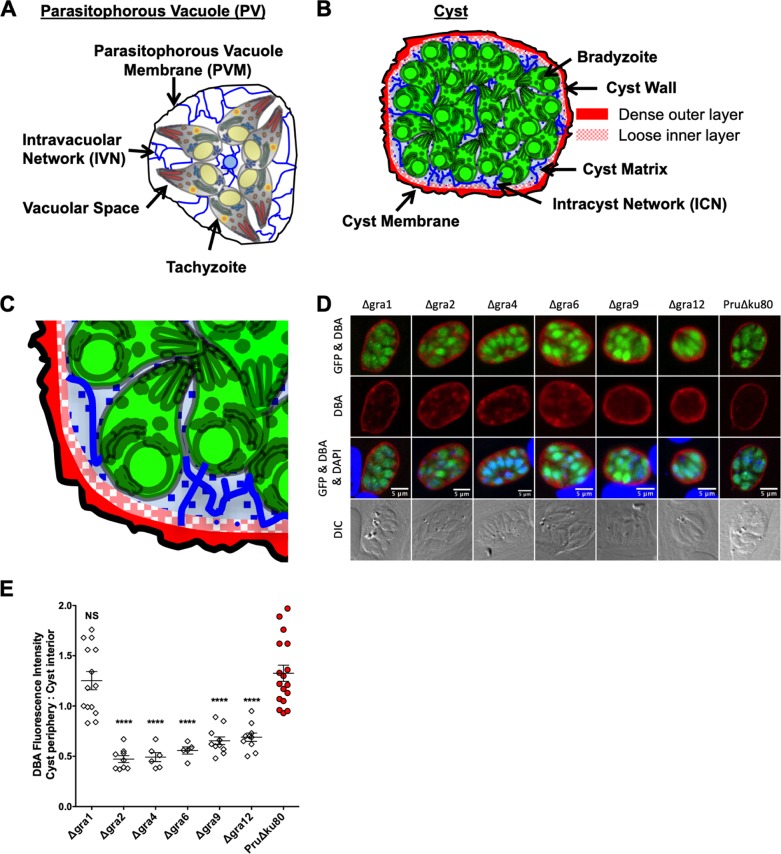
DBA staining intensity at the cyst periphery relative to the cyst interior is decreased in mutant strains that lack expression of IVN-associated GRA proteins, except for GRA1. (A) Schematic showing the primary features of the acute stage parasitophorous vacuole (PV). The parasitophorous vacuole membrane (PVM) (black circle) surrounds tachyzoites that are connected through the intravacuolar network (IVN) (blue lines). (B) Schematic showing the primary features of the chronic stage cyst. The cyst membrane (black circle) surrounds the cyst wall. The cyst wall is composed of a dense outer layer (solid red) and a less dense inner layer (dappled red). The space between the cyst wall and the bradyzoites is the cyst matrix, which contains matrix elements such as the intracyst network (ICN) (blue lines), filamentous materials, and vesicles. (C) Enlarged portion of the cyst wall showing the detail of the cyst wall composition. (D and E) *In vitro* cysts derived from different GRA deletion strains or parental PruΔ*ku80* were induced for 3 days. (D) Cysts were located using differential interference contrast (DIC) microscopy and imaged by confocal microscopy. The presence of bradyzoites inside cysts was verified by locating parasite nuclei with DAPI staining and verifying that each parasite nucleus was surrounded by expression of cytosolic GFP (GFP^+^ bradyzoites). Cysts were stained with DBA. The panels show GFP and DBA, DBA, GFP and DBA and DAPI, and DIC. Bars = 5 μm. (E) Cysts from each strain were analyzed to determine the ratio of Dolichos biflorus agglutinin (DBA) staining intensity at the cyst periphery relative to the cyst interior. Data were plotted as the average mean ratio of fluorescence intensity at the cyst periphery to the cyst interior ± standard error of the mean (SEM) (error bars) for each strain and was determined in 1 to 3 independent experiments: Δ*gra1* (one experiment; *n *= 14), Δ*gra2* (one experiment; *n *= 9), Δ*gra4* (one experiment; *n *= 6), Δ*gra6* (one experiment; *n *= 5), Δ*gra9* (one experiment; *n* = 11), Δ*gra12* (one experiment; *n *= 10), and PruΔ*ku80* (three experiments; *n *= 17). *P* values were calculated with a Student’s *t* test and indicated as follows: ****, *P* < 0.0001; NS, not significant.

The IVN in the tachyzoite-stage PV (Fig. 1A) resembles the intracyst network (ICN) of curved membrane tubules that link bradyzoites and the cyst wall (Fig. 1B). The current model of the cyst wall structure includes a dense outer cyst wall layer that lies beneath the limiting cyst membrane and a loose, less densely compacted, inner cyst wall layer that faces the cyst matrix (Fig. 1C) (19). Membrane tubules, filamentous materials, and vesicles have been visualized within the cyst wall (Fig. 1C) (19). To quantitatively assess development of the cyst wall, we developed a method to define the cyst wall region using a new Fiji macro (see Text S1 in the supplemental material), which measures fluorescence intensity of DBA parallel to the cyst wall, capturing the fluorescence intensity of each cyst layer (1 pixel thick) from outside the cyst wall, through the cyst wall region, and into the cyst matrix (Fig. S2). The cyst wall in cysts differentiated for longer than 1 day is six layers thick (Fig. S2). We recently reported that deletion of the IVN-associated GRA12 protein caused a defect in the rate of development of the cyst wall (47). To determine whether this defect was specific to GRA12 or was associated with other IVN-associated GRAs, we measured DBA fluorescence intensity at the cyst periphery of Δ*gra1*, Δ*gra2*, Δ*gra4*, Δ*gra6*, Δ*gra9*, and Δ*gra12* cysts (Fig. 1D), which reflects wall cargo (DBA-stained CST1) delivered to the cyst wall, compared to the cyst interior, which reflects wall cargo not yet delivered to the cyst wall (Fig. S3). For this quantitative analysis, the cyst periphery was defined as all of the layers of the cyst wall (Fig. S2) plus the two layers in the cyst matrix closest to the cyst wall to include cyst wall cargo that was already delivered beneath the cyst wall and most likely was actively being incorporated into the cyst wall (Fig. S3). The Δ*gra1* mutant exhibited a nearly identical ratio of DBA staining at the cyst periphery compared to the cyst interior as the parental PruΔ*ku80* strain (Fig. 1E). In contrast, Δ*gra2*, Δ*gra4*, Δ*gra6*, Δ*gra9*, and Δ*gra12* cysts exhibited a significant decrease in the DBA fluorescence intensity ratio (cyst periphery/cyst interior) (Fig. 1E).

10.1128/mSphere.00487-19.2 FIG S2 Defining the cyst wall and the locations of IVN-associated GRA proteins relative to the cyst wall strategy. (A to E) Schematic showing the locations of GRA proteins within the cyst wall. (A and B) Schematic showing the method of measurement. The mask (shown by the dotted blue line) was duplicated to form a region between two layers, approximately 1 pixel thick where the fluorescence intensity of that region was measured. (C to E) Infected HFFs on coverslips were treated with bradyzoite-inducing conditions for 7 days to induce *in vitro* cysts. Cysts were located using DIC microscopy and imaged by confocal microscopy. The presence of bradyzoites inside cysts was verified by locating parasite nuclei with DAPI staining (not shown) and verifying that each parasite nucleus was surrounded by expression of cytosolic GFP (GFP^+^ bradyzoites). Cysts were stained with DBA (shown in red), which highlights the cyst wall structure. Scale bars = 50 pixels. (C and D) The dotted lines (upper panels) highlight the region that is magnified below (lower panels). (C) The mask (shown in blue) is drawn using the fluorescence intensity of DBA (shown in red) (panel 1). The mask was duplicated to create a region between layers. Fourteen layers (L-2 to L-15) were measured from the mask outside the cyst to provide background readings. (D) Six layers (L-1 to L5) are the layers of the cyst wall region. (E) Representative layers of the cyst interior. Download FIG S2, TIF file, 0.6 MB. Copyright © 2019 Guevara et al. 2019 Guevara et al. This content is distributed under the terms of the Creative Commons Attribution 4.0 International license.

10.1128/mSphere.00487-19.3 FIG S3 Locations of IVN-associated GRAs. (A to D) Schematic illustration of the method used for quantification of the fluorescence intensity of GRA proteins within the cyst wall relative to the cyst interior. (A) A mask (shown by the blue line) is drawn using the fluorescence intensity of DBA (shown in red) to define the boundary of the cyst, which is named layer zero (L0). Scale bars = 50 pixels. (B) Schematic showing the primary features of the cyst and their definitions. The total fluorescence intensity of the cyst is one layer away from the mask, layer negative one (L-1). The fluorescence intensity of the cyst interior is layer five (L5) for day 1 or layer six (L6) for day 2 through day 10. The fluorescence intensity of the cyst periphery was determined by subtracting the fluorescence intensity of the cyst interior from the fluorescence intensity of the total cyst. (C and D) Infected HFFs on coverslips were treated with bradyzoite-inducing conditions for 7 days to induce *in vitro* cysts. Cysts were located using DIC microscopy and imaged by confocal microscopy. The presence of bradyzoites inside cysts was verified by locating parasite nuclei with DAPI staining (not shown) and verifying that each parasite nucleus was surrounded by expressed cytosolic GFP (GFP^+^ bradyzoites). Cysts were stained with DBA (shown in red), which highlights the cyst wall structure. The layers (L) (shown by the blue line) are denoted with a minus sign, which indicates layers outside the mask, or no sign, which indicates layers inside the mask. The total fluorescence intensity inside the blue line is measured at each layer. (C) Layers that compose the cyst wall. (D) Layers inside the cyst wall. Layer 5 (for day 1) or layer 6 (for day 2 through day 10) was determined to be the border between the cyst periphery, which includes the cyst wall plus two layers in the cyst matrix to account for proteins at the cyst periphery that are not yet incorporated into the cyst wall and the cyst interior (which includes everything within the cyst but excludes the cyst periphery). Download FIG S3, TIF file, 0.6 MB. Copyright © 2019 Guevara et al. 2019 Guevara et al. This content is distributed under the terms of the Creative Commons Attribution 4.0 International license.

10.1128/mSphere.00487-19.9 TEXT S1 Macro for location of T. gondii proteins within the cyst. Download Text S1, TXT file, 0.01 MB. Copyright © 2019 Guevara et al. 2019 Guevara et al. This content is distributed under the terms of the Creative Commons Attribution 4.0 International license.

### Localization of IVN-associated GRA proteins during *in vitro* development of the tachyzoite PV into mature cysts.

The ICN membrane tubules in the cyst wall and cyst matrix were previously hypothesized to originate from the IVN membranes of the tachyzoite-stage PV that developed into a cyst (19). In addition, IVN-associated GRA1, GRA2, GRA4, GRA6, GRA9, and GRA12 are highly expressed by tachyzoites, as well as by bradyzoites. We reasoned that if the ICN originates from the IVN, then the GRA proteins that localize with the IVN in the tachyzoite-stage PV would localize to both the cyst matrix and the cyst wall compartments in the developing cyst. We examined the localization of IVN-associated GRA proteins during *in vitro* cyst development using specific antibodies to locate GRA1, GRA2, GRA4, GRA6, and GRA9 and a Δ*gra12* mutant that was complemented with wild-type GRA12 tagged at the C terminus with hemagglutinin (HA) for visualization of GRA12 (Fig. S1D). We previously reported that cyst development and cyst burdens in mice were rescued after complementation of Δ*gra12* parasites with HA-tagged wild-type GRA12 (47). Tracking of GRA protein location(s) was performed using 6-h, 1-day, 2-day, 3-day, 7-day, and 10-day-old *in vitro* cysts after differentiation of the tachyzoite-stage PV.

### Six hours after differentiation of the tachyzoite-stage PV, IVN-associated GRAs are localized within the developing cyst.

Green fluorescent protein-positive (GFP^+^) bradyzoites were visible within the developing cyst (Fig. S4A), confirming previous findings that differentiation induced by alkaline switch rapidly upregulated expression of bradyzoite-stage genes, such as LDH2 and CST1 (53). DBA staining and GRA1, GRA2, GRA4, GRA6, GRA9, and GRA12 proteins were observed within the recently differentiated young cyst (Fig. S4A). DBA staining and these IVN-associated GRA proteins were observed toward the cyst periphery (Fig. S4A). Colocalization was occasionally seen between GRA2/GRA4 and GRA2/GRA9 (Fig. S4B). In contrast, GRA2/GRA6 and GRA2/GRA12 colocalization was observed in all cysts (Fig. S4B), and colocalization of GRA9 and GRA1 was observed toward the cyst periphery in all cysts (Fig. S4C).

**FIG 2:**
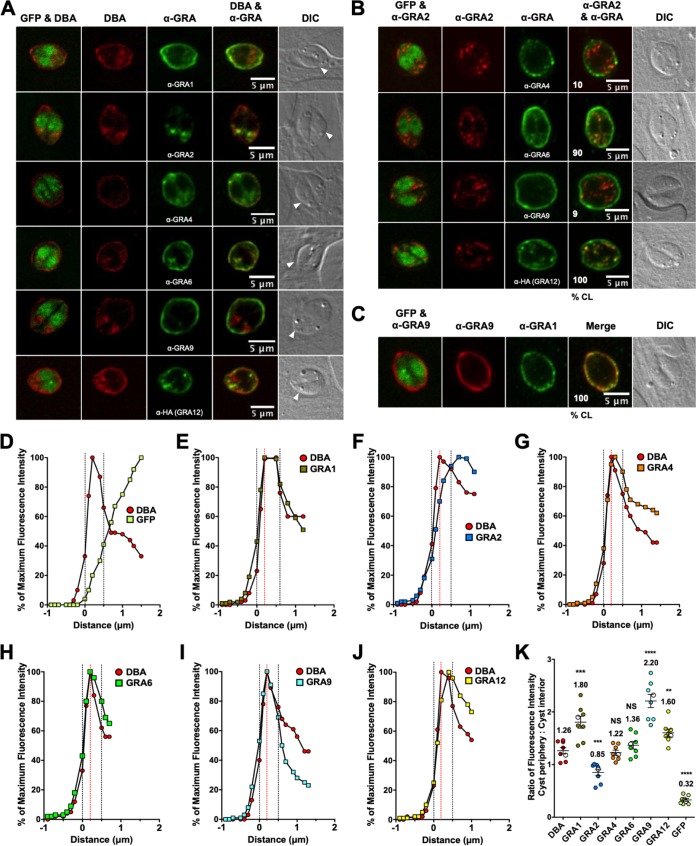
In 1-day cysts, IVN-associated GRAs, except for GRA2, are at the cyst periphery. Infected HFFs on coverslips were treated with bradyzoite-inducing conditions for 1 day to differentiate immature *in vitro* cysts. Cysts were located using DIC microscopy and imaged by confocal microscopy. The presence of bradyzoites inside cysts was verified by locating parasite nuclei with DAPI staining (not shown) and verifying that each parasite nucleus was surrounded by expressed cytosolic GFP (GFP^+^ bradyzoites). (A) Cysts were stained with DBA and anti-GRA antibody (α-GRA). For each GRA protein assessed, panels show GFP and DBA, DBA, α-GRA, DBA and α-GRA, and DIC (cyst wall indicated by white arrowheads). Bars = 5 μm. (B) GRA proteins were assessed for colocalization with GRA2. Cysts were stained with anti-GRA2 (red) and anti-GRA (green). The percentages of colocalization (% CL) of GRA2 for all imaged cysts were analyzed (shown in the bottom left corner of the α-GRA2 and α-GRA panels). Bars = 5 μm. (C) Cysts were stained with anti-GRA9 (red) and anti-GRA1 (green). The percentages of colocalization (% CL) of GRA9 and GRA1 for all imaged cysts were analyzed (shown in the bottom left corner of the merged panel). Bar = 5 μm. (D to J) Fluorescence intensity profiles of representative cysts shown in panel A were generated to quantify the location of GRA protein(s) relative to the cyst wall: DBA compared to GFP^+^ bradyzoites (D), DBA compared to GRA1 (E), DBA compared to GRA2 (F), DBA compared to GRA4 (G), DBA compared to GRA6 (H), DBA compared to GRA9 (I), and DBA compared to GRA12 (J). The dotted black vertical lines define the cyst wall region. The dotted red vertical line indicates the middle of the cyst wall, which corresponds to the peak DBA fluorescence intensity. (K) Fluorescence intensities of DBA, GRA1, GRA2, GRA4, GRA6, GRA9, GRA12, and GFP were measured at the cyst periphery and within the cyst (cyst interior). Data plotted are the mean ratio of fluorescence intensity at the cyst periphery to the cyst interior ± SEM (*n *= 8 cysts). Cyst images shown in panel A correspond to the cyst near the mean (shown as white circles) for each GRA protein. The numerical ratio for the mean fluorescence intensity is labeled for each GRA protein and the DBA control. *P* values were calculated with a Student’s *t* test and indicated as follows: **, *P* < 0.01; ***, *P* < 0.005; ****, *P* < 0.0001; NS, not significant.

**FIG 3:**
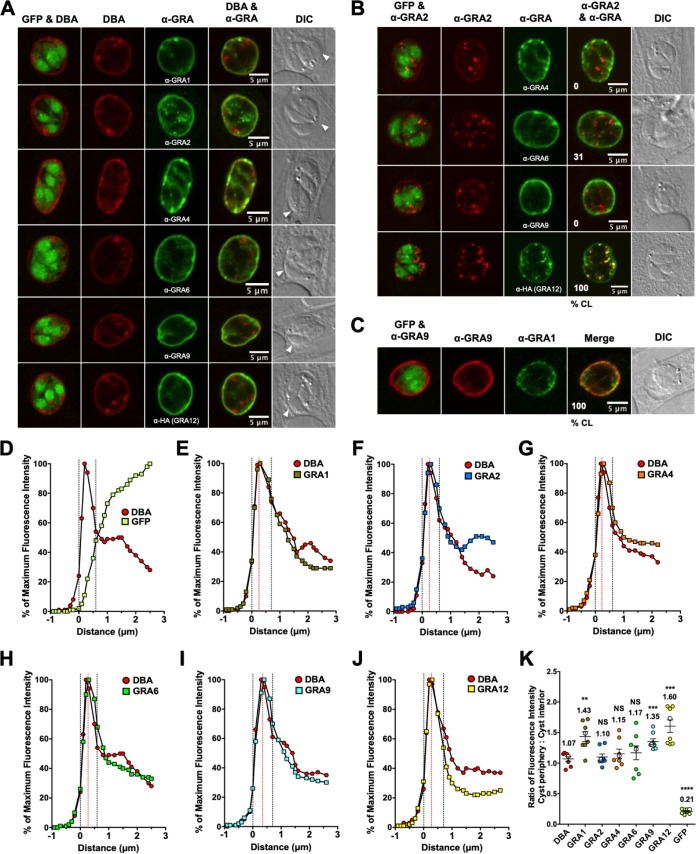
In 2-day cysts, GRA2 transitions to the cyst periphery. Infected HFFs on coverslips were treated with bradyzoite-inducing conditions for 2 days to differentiate immature *in vitro* cysts. Cysts were located using DIC microscopy and imaged by confocal microscopy. The presence of bradyzoites inside cysts was verified by locating parasite nuclei with DAPI staining (not shown) and verifying that each parasite nucleus was surrounded by expressed cytosolic GFP (GFP^+^ bradyzoites). (A) Cysts were stained with DBA and anti-GRA antibody (α-GRA). For each GRA protein assessed, panels show GFP and DBA, DBA, α-GRA, DBA and α-GRA, and DIC (cyst wall indicated by white arrowheads). Bars = 5 μm. (B) GRA proteins were assessed for colocalization with GRA2. Cysts were stained with α-GRA2 (red) and α-GRA (green). The percentages of colocalization (% CL) of GRA2 for all imaged cysts were analyzed (shown in the bottom left corner of the α-GRA2 and α-GRA panels). Bars = 5 μm. (C) Cysts were stained with α-GRA9 (red) and α-GRA1 (green). The percentages of colocalization (% CL) of GRA9 and GRA1 for all imaged cysts were analyzed (bottom left corner of the merged panel). Bar = 5 μm. (D to J) Fluorescence intensity profiles of representative cysts shown in panel A were generated to quantify the location of GRA protein(s) relative to the cyst wall as follows: DBA compared to GFP^+^ bradyzoites (D), DBA compared to GRA1 (E), DBA compared to GRA2 (F), DBA compared to GRA4 (G), DBA compared to GRA6 (H), DBA compared to GRA9 (I), and DBA compared to GRA12 (J). The dotted black vertical lines define the cyst wall region. The dotted red vertical line indicates the middle of the cyst wall, which corresponds to the peak DBA fluorescence intensity. (K) Fluorescence intensities of DBA, GRA1, GRA2, GRA4, GRA6, GRA9, GRA12, and GFP were measured at the cyst periphery and within the cyst (cyst interior). Data plotted are the mean ratio of fluorescence intensity at the cyst periphery to the cyst interior ± SEM (*n *= 8 cysts). Cyst images shown in panel A correspond to the cyst near the mean (shown as white circles) for each GRA protein. The numerical ratio for the mean fluorescence intensity is labeled for each GRA protein and the DBA control. *P* values were calculated with a Student’s *t* test and indicated as follows: **, *P* < 0.01; ***, *P* < 0.005; ****, *P* < 0.0001; NS, not significant.

**FIG 4:**
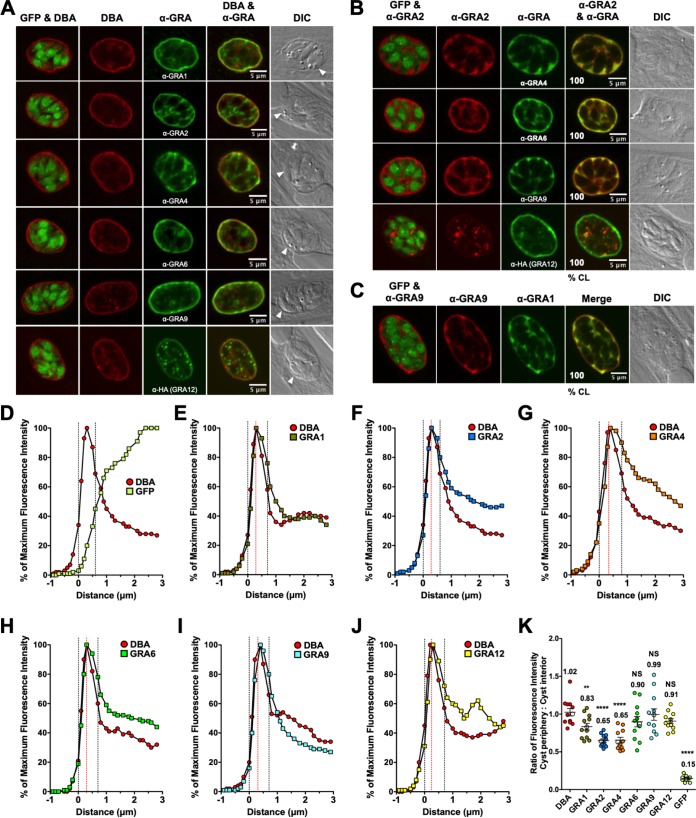
In 3-day cysts, IVN-associated GRAs localize to the developing cyst wall and to the cyst matrix. Infected HFFs on coverslips were treated with bradyzoite-inducing conditions for 3 days to differentiate immature *in vitro* cysts. Cysts were located using DIC microscopy and imaged by confocal microscopy. The presence of bradyzoites inside cysts was verified by locating parasite nuclei with DAPI staining (not shown) and verifying that each parasite nucleus was surrounded by expressed cytosolic GFP (GFP^+^ bradyzoites). (A) Cysts were stained with DBA and anti-GRA antibody (α-GRA). For each GRA protein assessed, panels show GFP and DBA, DBA, α-GRA, DBA and α-GRA, and DIC (cyst wall indicated by white arrowheads). Bars = 5 μm. (B) GRA proteins were assessed for colocalization with GRA2. Cysts were stained with anti-GRA2 (red) and anti-GRA (green). The percentages of colocalization (% CL) of GRA2 for all imaged cysts were analyzed (bottom left corner of the α-GRA2 and α-GRA panels). Bars = 5 μm. (C) Cysts were stained with anti-GRA9 (red) and anti-GRA1 (green). The percentages of colocalization (% CL) of GRA9 and GRA1 for all imaged cysts were analyzed (bottom left corner of the merged panel). Bar = 5 μm. (D to J) Fluorescence intensity profiles of representative cysts shown in panel A were generated to quantify the location of GRA protein(s) relative to the cyst wall: (D) DBA compared to GFP^+^ bradyzoites (D), DBA compared to GRA1 (E), DBA compared to GRA2 (F), DBA compared to GRA4 (G), DBA compared to GRA6 (H), DBA compared to GRA9 (I), and DBA compared to GRA12 (J). The dotted black vertical lines define the cyst wall region. The dotted red vertical line indicates the middle of the cyst wall, which corresponds to the peak DBA fluorescence intensity. (K) Fluorescence intensities of DBA, GRA1, GRA2, GRA4, GRA6, GRA9, GRA12, and GFP were measured at the cyst periphery and within the cyst (cyst interior). Data plotted are the mean ratio of fluorescence intensity at the cyst periphery to the cyst interior ± SEM (*n *= 8 cysts). Cyst images shown in panel A correspond to the cyst near the mean (shown as white circles) for each GRA protein. The numerical ratio for the mean fluorescence intensity is labeled for each GRA protein and the DBA control. *P* values were calculated with a Student’s *t* test and indicated as follows: **, *P* < 0.01; ****, *P* < 0.0001; NS, not significant.

10.1128/mSphere.00487-19.4 FIG S4 In 6-h cysts, IVN-associated GRAs are localized to the cyst periphery shortly after differentiation while GRA2 molecules are delayed to the cyst periphery. Infected HFFs on coverslips were treated with bradyzoite-inducing conditions for 6 hours to differentiate *in vitro* cysts. Cysts were located using DIC microscopy and imaged by confocal microscopy. The presence of a bradyzoite inside a cyst was verified by locating parasite nuclei with DAPI staining (not shown) and verifying each parasite nucleus was surrounded by expression of cytosolic GFP (GFP^+^ bradyzoites). (A) Cysts were stained with DBA and anti-GRA antibody (αGRA). For each GRA protein assessed, panels show GFP and DBA, αGRA, DBA and αGRA, and DIC. Scale bars = 5 μm. (B) GRA proteins were assessed for colocalization with GRA2. Cysts were stained with α-GRA2 (red) and α-GRA (green). Scale bars = 5 μm. (C) Cysts were stained with α-GRA9 (red) and α-GRA1 (green). Scale bars = 5 μm. Fluorescence intensity profiles of the cysts in Fig. 2B (day 1) (D to H), Fig. 3B (day 2) (I to M), and Fig. 4B (day 3) (N to R) were generated to visualize the locations of proteins compared to the IVN-associated GRA2 protein. The dotted black lines define the cyst wall region. (D to R) GRA2 compared to GRA4, GRA6, GRA9, and GRA12. Fluorescence intensity profiles of the cysts in Fig. 2C (day 1) (H), Fig. 3C (day 2) (M), and Fig. 4C (day 3) (R) were generated to visualize the location of GRA9 compared to GRA1. Download FIG S4, TIF file, 1.9 MB. Copyright © 2019 Guevara et al. 2019 Guevara et al. This content is distributed under the terms of the Creative Commons Attribution 4.0 International license.

### In immature 1-day cysts, IVN-associated GRAs, except for GRA2, are at the cyst periphery.

GFP^+^ bradyzoites were visible within a DBA-stained cyst (Fig. 2A). DBA-stained puncta were observed in the cyst matrix, indicating that the cyst wall was actively being constructed (Fig. 2A). All GRAs except for GRA2 were localized to the cyst periphery (Fig. 2A). Puncta of GRA2 were observed within the cyst matrix (Fig. 2A). Staining of dense granules inside the GFP^+^ region defining bradyzoites was not readily apparent (Fig. 2), suggesting that the dense granules were not significantly stained after permeabilization of cysts in 0.2% Triton X-100. Colocalization was rarely observed between GRA2/GRA4 or GRA2/GRA9 within the cyst matrix (Fig. 2B). In contrast, bright puncta of GRA2/GRA6 and GRA2/GRA12 were colocalized within the cyst matrix in nearly all cysts (Fig. 2B), and GRA9 and GRA1 were colocalized in the cyst matrix and at the cyst periphery in all cysts (Fig. 2C).

To quantitatively assess the location(s) of GRA proteins in the cyst, we measured the cyst fluorescence intensity profiles for GRA proteins, DBA, and GFP using the Fiji macro (Text S1) to measure fluorescence intensity of each cyst layer (1 pixel thick) from outside the cyst wall, through the cyst wall region, and into the cyst matrix (Fig. S3). To define the cyst wall region relative to the cyst matrix or outside the cyst wall region, the fluorescence intensity of DBA and GFP was measured using the rationale that (i) DBA selectively stains the cyst wall, (ii) DBA does not stain the host cell, (iii) bradyzoites can touch the cyst wall but are not part of the cyst wall structure, and (iv) GFP expressed by bradyzoites is present in the bradyzoite cytosol (see Materials and Methods). In 1-day cysts, the cyst wall region was defined by five layers (Fig. 2D). The fluorescence intensity peaks of GRA1, GRA4, GRA6, GRA9, and GRA12, but not GRA2, overlapped with the DBA fluorescence intensity peak, indicating their presence at the cyst periphery (Fig. 2E to J). For each GRA, we measured the fluorescence intensity at the cyst periphery compared to the cyst interior and compared it to DBA (Fig. 2K). In comparison to DBA, GRA1, GRA9, and GRA12 exhibited a significant increase in fluorescent intensity at the cyst periphery relative to the cyst interior (Fig. 2K), which revealed that these GRA molecules were more prominent at the cyst periphery on day 1. In contrast, GRA2 exhibited a significant decrease in fluorescent intensity at the cyst periphery relative to the cyst interior, which revealed that GRA2 was more prominent in the cyst interior (Fig. 2K). GRA4 and GRA6 exhibited similar ratios in fluorescent intensity to DBA (Fig. 2K). In addition, the GRA2 fluorescence intensity peak did not align with the GRA4, GRA6, GRA9, or GRA12 peaks that were located at the cyst periphery, confirming that GRA2 was not present at the cyst periphery (Fig. S4D to G). In contrast, GRA9 and GRA1 peaks were located at the cyst periphery (Fig. S4H), confirming colocalization of GRA9 and GRA1 (Fig. 2C).

### In immature 2-day cysts, GRA2 transitions to the cyst periphery.

DBA-stained puncta were observed in the cyst matrix, indicating that the cyst wall was actively being constructed (Fig. 3A). GRA1, GRA2, GRA4, GRA6, GRA9, and GRA12 were localized to the cyst periphery in 2-day cysts (Fig. 3A). Puncta of GRA2 were observed within the cyst matrix (Fig. 3A and B). While weak colocalization was observed between GRA2/GRA6 within the cyst matrix, GRA2/GRA4 and GRA2/GRA9 were not colocalized (Fig. 3B). In contrast, bright puncta of GRA2 and GRA12 were colocalized within the cyst matrix in all cysts (Fig. 3B). All GRAs were present at the cyst periphery (Fig. S4I to L). However, relative to GRA4, GRA6, GRA9, and GRA12, the GRA2 peak was shifted to the right (toward the cyst matrix), indicating that GRA2 was still prominent in the cyst matrix and was still actively transitioning toward the cyst wall (Fig. S4I to L). Colocalization between GRA9 and GRA1 was observed in the cyst matrix and at the cyst periphery in all cysts (Fig. 3C and Fig. S4M).

In contrast to five layers of the cyst wall region observed in 1-day cysts, the cyst wall region in 2-day cysts occupied six layers (Fig. 3D to J). The fluorescence intensity peaks of all IVN-associated GRAs overlapped with the DBA fluorescence intensity peak, indicating their presence at the cyst periphery (Fig. 3E to J). In comparison to DBA, GRA1, GRA9, and GRA12 exhibited a significant increase in fluorescent intensity at the cyst periphery relative to the cyst interior (Fig. 3K), which revealed that these GRA molecules were more prominent at the cyst periphery. In contrast, GRA2, GRA4, and GRA6 exhibited similar fluorescent intensity ratios to DBA, which revealed that these GRA molecules were prominent in a nearly equal ratio at the cyst periphery and in the cyst interior (Fig. 3K).

### In immature 3-day cysts, IVN-associated GRAs localize to the developing cyst wall and to the cyst matrix.

Similar to 1-day cysts (Fig. 2) or 2-day cysts (Fig. 3), DBA still prominently stained the cyst matrix of 3-day cysts (Fig. 4A to C), indicating that the cyst wall was being actively built at an accelerated rate and suggesting that 3-day cysts were immature. All GRAs were localized to the cyst matrix and to the cyst wall (Fig. 4A). Colocalization between GRA2/GRA4, GRA2/GRA6, and GRA2/GRA9 was observed in the cyst matrix and at the cyst periphery in all cysts (Fig. 4B), and the intensity peaks of GRA4, GRA6, and GRA9 overlapped with GRA2 (Fig. S4N to P). GRA2/GRA4, GRA2/GRA6, and GRA2/GRA9 were colocalized in the cyst matrix in the tight spaces between the bradyzoites, which were enlarged into triangular spaces near the cyst periphery (Fig. 4B). In contrast, GRA2 and GRA12 were colocalized in bright puncta in the cyst matrix in all cysts (Fig. 4B). The intensity peaks of GRA2 and GRA12 did not overlap in the cyst wall region (Fig. S4Q). Colocalization between GRA9 and GRA1 was observed in the cyst matrix and within the cyst wall in all cysts (Fig. 4C and Fig. S4R).

The cyst wall region in 3-day cysts occupied six layers (Fig. 4D to J). The fluorescence intensity peaks of all IVN-associated GRAs overlapped with the DBA fluorescence intensity peak, indicating their presence at the cyst wall (Fig. 4E to J). In comparison to DBA, GRA1, GRA2, and GRA4 exhibited significant decreases in fluorescent intensity at the cyst periphery relative to the cyst interior (Fig. 4K), which revealed that these GRA molecules were also prominent within the cyst matrix. In contrast, GRA6, GRA9, and GRA12 exhibited similar fluorescent intensity ratios to DBA, which revealed that these GRA molecules were prominent in approximately equal ratios at the cyst periphery and in the cyst interior (Fig. 4K).

### In mature 7-day cysts, IVN-associated GRAs localize to different layers in the cyst wall and in different patterns in the cyst matrix.

**FIG 5:**
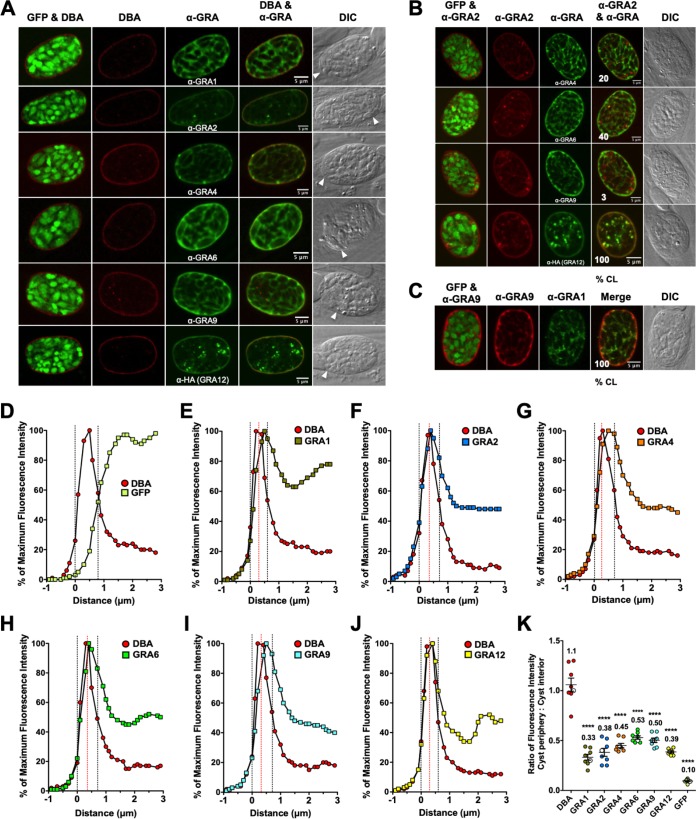
In 7-day cysts, IVN-associated GRAs localize to different layers in the cyst wall and in different patterns in the cyst matrix. Infected HFFs on coverslips were treated with bradyzoite-inducing conditions for 7 days to differentiate mature *in vitro* cysts. Cysts were located using DIC microscopy and imaged by confocal microscopy. The presence of bradyzoites inside cysts was verified by locating parasite nuclei with DAPI staining (not shown) and verifying that each parasite nucleus was surrounded by expressed cytosolic GFP (GFP^+^ bradyzoites). (A) Cysts were stained with DBA and anti-GRA antibody (α-GRA). For each GRA protein assessed, panels show GFP and DBA, DBA, α-GRA, DBA and α-GRA, and DIC (cyst wall indicated by white arrowheads). Bars = 5 μm. (B) GRA proteins were assessed for colocalization with GRA2. Cysts were stained with anti-GRA2 (red) and anti-GRA (green). The percentages of colocalization (% CL) of GRA2 for all imaged cysts were analyzed (bottom left corner of the α-GRA2 and α-GRA panels). Bars = 5 μm. (C) Cysts were stained with anti-GRA9 (red) and anti-GRA1 (green). The percentages of colocalization (% CL) of GRA9 and GRA1 for all imaged cysts were analyzed (bottom left corner of the merged panel). Bar = 5 μm. (D to J) Fluorescence intensity profiles of representative cysts shown in panel A were generated to quantify the location of GRA protein(s) relative to the cyst wall: DBA compared to GFP^+^ bradyzoites (D), DBA compared to GRA1 (E), DBA compared to GRA2 (F), DBA compared to GRA4 (G), DBA compared to GRA6 (H), DBA compared to GRA9 (I), and DBA compared to GRA12 (J). The dotted black vertical lines define the cyst wall region. The dotted red vertical line indicates the middle of the cyst wall, which corresponds to the peak DBA fluorescence intensity. (K) Fluorescence intensities of DBA, GRA1, GRA2, GRA4, GRA6, GRA9, GRA12, and GFP were measured at the cyst periphery and within the cyst (cyst interior). Data plotted are the mean ratio of fluorescence intensity at the cyst periphery to the cyst interior ± SEM (*n *= 8 cysts). Cyst images shown in panel A correspond to the cyst near the mean (shown as the white circles) for each GRA protein. The numerical ratio for the mean fluorescence intensity is labeled for each GRA protein and the DBA control. *P* values were calculated with a Student’s *t* test and indicated as follows: ****, *P* < 0.0001.

All GRAs were localized to the cyst matrix; GRA1, GRA4, GRA6, and GRA9 occupied the spaces between the bradyzoites, while bright puncta of GRA2 and GRA12 were visible within the cyst matrix (Fig. 5A and Fig. S5A). All GRAs also localized to the cyst wall (Fig. 5A and Fig. S5A). Colocalization between GRA2 and GRA12 was observed as bright puncta in the cyst matrix and in the cyst wall in all cysts (Fig. 5B). Consistent with their colocalization in the cyst wall (Fig. 5B), the intensity peaks of GRA2 and GRA12 overlapped (Fig. S5E). GRA2 and GRA6 were colocalized within the cyst matrix in 40% of cysts, and GRA2/GRA4 and GRA2/GRA9 were rarely colocalized within the cyst matrix (Fig. 5B). GRA4, GRA6, GRA9, and GRA12 intensity peaks overlapped with GRA2 at the cyst wall (Fig. S5B to E). Colocalization of GRA9 and GRA1 was observed within the cyst matrix and the cyst wall in all cysts (Fig. 5C and Fig. S5F).

**FIG 6:**
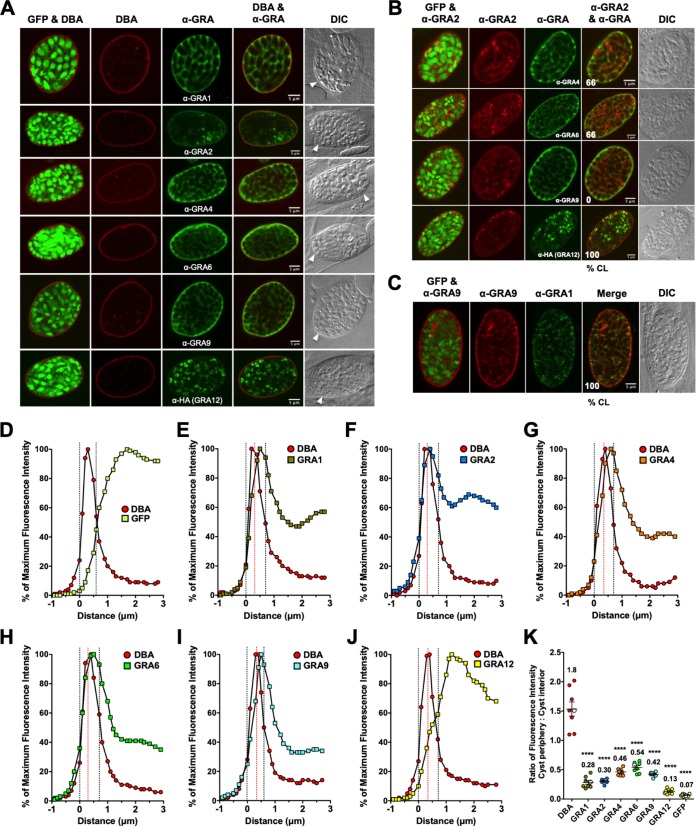
In 10-day cysts, IVN-associated GRAs are localized in the cyst wall, except for GRA12, and are localized throughout the cyst matrix. Infected HFFs on coverslips were treated with bradyzoite-inducing conditions for 10 days to differentiate mature *in vitro* cysts. Cysts were located using DIC microscopy and imaged by confocal microscopy. The presence of bradyzoites inside cysts was verified by locating parasite nuclei with DAPI staining (not shown) and verifying that each parasite nucleus was surrounded by expressed cytosolic GFP (GFP^+^ bradyzoites). (A) Cysts were stained with DBA and anti-GRA antibody (α-GRA). For each GRA protein assessed, panels show GFP and DBA, DBA, α-GRA, DBA and α-GRA, and DIC (cyst wall indicated by white arrowheads). Bars = 5 μm. (B) GRA proteins were assessed for colocalization with GRA2. Cysts were stained with anti-GRA2 (red) and anti-GRA (green). The percentages of colocalization (% CL) of GRA2 for all imaged cysts were analyzed (bottom left corners of the α-GRA2 and α-GRA panels). Bars = 5 μm. (C) Cysts were stained with anti-GRA9 (red) and anti-GRA1 (green). The percentage of colocalization (% CL) of GRA9 and GRA1 for all imaged cysts were analyzed (bottom left corner of the merged panel). Bar = 5 μm. (D to J) Fluorescence intensity profiles of representative cysts shown in panel A were generated to quantify the location of GRA protein(s) relative to the cyst wall: DBA compared to GFP^+^ bradyzoites (D), DBA compared to GRA1 (E), DBA compared to GRA2 (F), DBA compared to GRA4 (G), DBA compared to GRA6 (H), DBA compared to GRA9 (I), and DBA compared to GRA12 (J). The dotted black vertical lines define the cyst wall region. The dotted red vertical line indicates the middle of the cyst wall, which corresponds to the peak DBA fluorescence intensity. (K) Fluorescence intensities of DBA, GRA1, GRA2, GRA4, GRA6, GRA9, GRA12, and GFP were measured at the cyst periphery and within the cyst (cyst interior). Data plotted are the mean ratio of fluorescence intensity at the cyst periphery to the cyst interior ± SEM (*n *= 8 cysts). Cyst images shown in panel A correspond to the cyst near the mean (shown as white circles) for each GRA protein. The numerical ratio for the mean fluorescence intensity is labeled for each GRA protein and the DBA control. *P* values were calculated with a Student’s *t* test and indicated as follows: ****, *P* < 0.0001.

10.1128/mSphere.00487-19.5 FIG S5 GRA2 and GRA12 localize at the cyst wall in 7-day and 10-day cysts, and then GRA12 leaves the cyst wall in 10-day cysts. (A) Images from Fig. 5A were magnified to show the GRAs (shown in green) relative to the cyst wall stained by DBA (shown in red). Scale bars = 2 μm. (B to E) Fluorescence intensity profiles of the cysts in Fig. 5B were generated to visualize the locations of proteins compared to the IVN-associated GRA2 protein. (B to E) GRA2 compared to GRA4 (B), GRA6 (C), GRA9 (D), and GRA12 (E). (F) Fluorescence intensity profiles of the cysts in Fig. 5C were generated to visualize the location of GRA9 compared to GRA1. (B to F) The dotted black lines define the cyst wall region as defined in the legend to Fig. 5D. (G) Images from Fig. 6A were magnified to show the GRAs (shown in green) relative to the cyst wall as stained by DBA (shown in red). Scale bars = 2 μm. (H to L) Fluorescence intensity profiles of the cysts in Fig. 6B were generated to visualize the locations of proteins compared to the IVN-associated GRA2 protein. (H to L) GRA2 compared to GRA4 (H), GRA6 (I), GRA9 (J), and GRA12 (K). (L) Fluorescence intensity profiles of the cysts in Fig. 6C were generated to visualize the location of GRA9 compared to GRA1. (H to L) The dotted black lines define the cyst wall region as defined in the legend to Fig. 6D. Download FIG S5, TIF file, 0.5 MB. Copyright © 2019 Guevara et al. 2019 Guevara et al. This content is distributed under the terms of the Creative Commons Attribution 4.0 International license.

The cyst wall region in 7-day cysts occupied six layers (Fig. 5D to J). The fluorescence intensity peaks of all IVN-associated GRAs overlapped with the DBA fluorescence intensity peak (Fig. 5E to J). The intensity peaks of GRA2 (Fig. 5F), GRA6 (Fig. 5H), and GRA12 (Fig. 5J) were similar to the peak of DBA, suggesting the presence of GRA2, GRA6, and GRA12 throughout the cyst wall. In contrast, the intensity peaks of GRA1 (Fig. 5E), GRA4 (Fig. 5G), and GRA9 (Fig. 5I) were shifted to the right (toward the cyst interior) compared to the DBA peak, indicating their presence in the less dense inner layer of the cyst wall. In comparison to DBA, GRA1, GRA2, GRA4, GRA6, GRA9, and GRA12 exhibited significant decreases in fluorescent intensity at the cyst periphery relative to the cyst interior (Fig. 5K), and this revealed that these GRA molecules were also prominent in the cyst interior.

### In mature 10-day cysts, IVN-associated GRAs are localized in the cyst wall, except for GRA12, and are localized throughout the cyst matrix.

All GRAs localized to the cyst matrix; GRA1, GRA4, GRA6, and GRA9 occupied the spaces between the bradyzoites, while bright puncta of GRA2 and GRA12 were prominent within the cyst matrix (Fig. 6A). GRA12 was the only GRA not localized to the cyst wall (Fig. 6A and Fig. S5G). In many cysts, GRA2/GRA4 and GRA2/GRA6 were colocalized in the cyst matrix and the cyst wall (Fig. 6B), and their intensity peaks overlapped in the cyst wall (Fig. S5H and I). GRA2/GRA9 were not colocalized in 10-day cysts (Fig. 6B and Fig. S5J). Bright puncta containing GRA2 and GRA12 were colocalized within the cyst matrix in all cysts (Fig. 6B), though GRA12 was not observed in the cyst wall (Fig. 6B and Fig. S5K). Colocalization of GRA9 and GRA1 was observed within the cyst matrix and in the cyst wall in all cysts (Fig. 6C and Fig. S5L).

The cyst wall region of 10-day cysts occupied six layers (Fig. 6D to J). The fluorescence intensity peaks of all IVN-associated GRAs, except for GRA12, overlapped with the DBA fluorescence intensity peak (Fig. 6E to J). GRA12 was not detected in the cyst wall in 10-day cysts. The intensity peaks of GRA2 (Fig. 6F) and GRA6 (Fig. 6H) were similar to that of DBA, indicating the presence of GRA2 and GRA6 throughout the cyst wall. In contrast, the intensity peaks of GRA1 (Fig. 6E), GRA4 (Fig. 6G), and GRA9 (Fig. 6I) were shifted toward the right, to the cyst interior, compared to the DBA peak, indicating their increased presence in the less dense inner layer of the cyst wall (19). For each GRA, we measured the fluorescence intensity at the cyst periphery compared to the cyst interior and compared it to DBA (Fig. 6K). In comparison to DBA, GRA1, GRA2, GRA4, GRA6, GRA9, and GRA12 exhibited significant decreases in fluorescent intensity at the cyst periphery relative to the cyst interior (Fig. 6K), which revealed that all of these GRA proteins were prominent in the cyst interior.

### GRA2 expression controls DBA staining intensity at the cyst periphery relative to the cyst interior.

IVN-associated GRA2, GRA4, GRA6, GRA9, and GRA12 mutants exhibited significant defects in the DBA fluorescent intensity at the cyst periphery relative to the cyst interior in 3-day cysts (Fig. 1D and E). To further assess this defect, we evaluated the development of Δ*gra2* cysts. GFP^+^ bradyzoites were visible within DBA-stained Δ*gra2* cysts that lacked expression of GRA2 (Fig. S6A to E). Compared to parental PruΔ*ku80* cysts, DBA stain was more prominently observed within the Δ*gra2* cyst matrix in 7-day and 10-day cysts (Fig. S6D and E). To quantify the location of DBA, we measured DBA fluorescence intensity at the cyst periphery relative to the cyst interior. Notably, in 1-day cysts, there was no difference in the DBA fluorescence intensity ratio of Δ*gra2* cysts in comparison to parental PruΔ*ku80* cysts (Fig. S6F). However, 2-day to 10-day Δ*gra2* cysts exhibited a significant decrease in the DBA fluorescence intensity ratio (cyst periphery/cyst interior) (Fig. S6F), suggesting that GRA2 expression was required by day 2 of cyst development for normal maturation of the cyst wall. We also measured cyst area of 3-day, 7-day, and 10-day Δ*gra2* and PruΔ*ku80* cysts to determine whether the deletion of GRA2 affected overall cyst size. Deletion of GRA2 had no significant effect on cyst size (Fig. S6G), and this suggested that the overall surface area of the cyst wall was not affected.

10.1128/mSphere.00487-19.6 FIG S6 GRA2 controls DBA staining intensity at the cyst periphery relative to the cyst interior. (A to E) HFFs on coverslips were infected with PruΔ*ku80* or PruΔ*ku80*Δ*gra2* (Δ*gra2*) and treated with bradyzoite-inducing conditions to differentiate *in vitro* cysts for 1 day (A), 2 days (B), 3 days (C), (D) 7 days, or 10 days (E). Cysts were located using DIC microscopy and imaged by confocal microscopy. The presence of bradyzoites inside cysts was verified by locating parasite nuclei with DAPI staining (not shown) and verifying that each parasite nucleus was surrounded by expressed cytosolic GFP (GFP^+^ bradyzoites shown in the first panel of panels A to E). Cysts were stained with DBA (red) and α-GRA2 (green). Representative cysts are shown. Scale bars = 5 μm. (F) The fluorescence intensity of DBA was measured at the cyst periphery and within the cyst (cyst interior) for each time point. Data plotted are the mean ratio of fluorescence intensity at the cyst periphery to cyst interior ± SEM (*n *= 8 cysts for all times other than 3 days; for day 3, PruΔ*ku80*, *n *= 25 cysts, and Δ*gra2*, *n *= 17 cysts). *P* values were calculated with a Student’s *t* test and indicated as follows: *, *P* < 0.05; ***, *P* < 0.005; ****, *P* < 0.0001; NS, not significant. (G) Cyst area ± SEM (*n *= 24 cysts) was measured at the indicated times postdifferentiation. *P* values were calculated with a Student’s *t* test (NS, not significant). Download FIG S6, TIF file, 2.0 MB. Copyright © 2019 Guevara et al. 2019 Guevara et al. This content is distributed under the terms of the Creative Commons Attribution 4.0 International license.

### GRA2 expression is required for organization of the cyst matrix and the localization of GRA4 and GRA6 at the cyst periphery.

**FIG 7:**
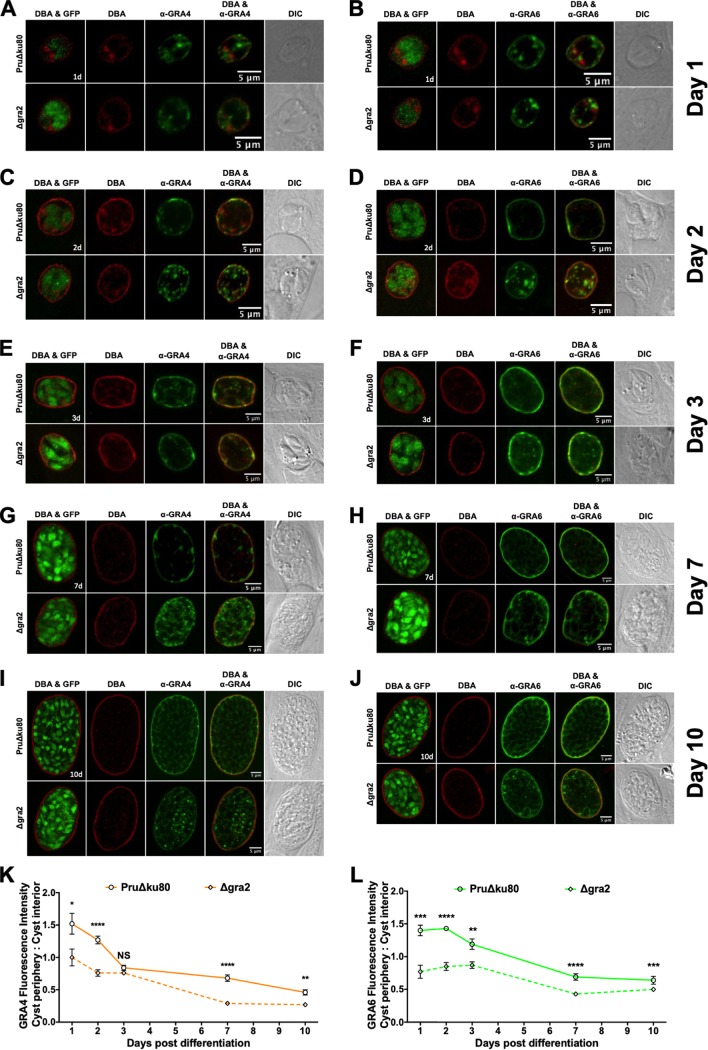
GRA2 controls the localization of GRA4 and GRA6 at the cyst periphery and the organization of the cyst matrix. (A to J) HFFs on coverslips were infected with PruΔ*ku80* or PruΔ*ku80*Δ*gra2* (Δ*gra2*) and treated with bradyzoite-inducing conditions to differentiate *in vitro* cysts for 1 day (A and B), 2 days (C and D), 3 days (E and F), 7 days (G and H), or 10 days (I and J). Cysts were located using DIC microscopy and imaged by confocal microscopy. The presence of bradyzoites inside cysts was verified by locating parasite nuclei with DAPI staining (not shown) and verifying that each parasite nucleus was surrounded by expressed cytosolic GFP (GFP^+^ bradyzoites shown in the leftmost images of panels A to J). Cysts were stained with DBA (red) and anti-GRA4 antibody (α-GRA4) (green) or with DBA (red) and α-GRA6 (green) as indicated. Representative cysts are shown. Bars = 5 μm. (K and L) Fluorescence intensity of GRA4 (K) or GRA6 (L) was measured at the cyst periphery and within the cyst (cyst interior) for each time point. Data plotted are the mean ratio of fluorescence intensity at the cyst periphery to cyst interior ± SEM (*n *= 8 cysts). *P* values were calculated with a Student’s *t* test and indicated as follows: *, *P* < 0.05; **, *P* < 0.01; ***, *P* < 0.005; ****, *P* < 0.0001; NS, not significant.

On the basis of the defect in DBA accumulation at the periphery relative to the cyst interior in Δ*gra2* cysts, we evaluated the localization of GRA4 and GRA6 in Δ*gra2* and PruΔ*ku80* cysts. GRA4 and GRA6 were observed at the cyst wall (Fig. 7A to J and Fig. S7A to J). However, notable differences in the localization of GRA4 and GRA6 were observed in Δ*gra2* cysts compared to PruΔ*ku80* cysts (Fig. 7A to J). In Δ*gra2* cysts, GRA4 and GRA6 were more readily observed as puncta inside the cyst matrix and the cyst matrix pattern was absent, suggesting that the cyst matrix was disorganized (Fig. 7A to J). To quantify the locations of GRA4 and GRA6, the fluorescence intensities of DBA and GRAs were measured in PruΔ*ku80* and Δ*gra2* cysts. The fluorescent intensity profiles of GRA4 and GRA6 were increased in the cyst interior in Δ*gra2* cysts (Fig. S7A to J). Correspondingly, 1-day to 10-day Δ*gra2* cysts exhibited a significant decrease in the fluorescent intensity ratio (cyst periphery/cyst interior) of GRA4 and GRA6 (Fig. 7K and L). These results suggested that GRA2 expression controls the relative rate of accumulation of other GRA proteins in the developing cyst wall.

10.1128/mSphere.00487-19.7 FIG S7 GRA2 controls the localization of GRA4 and GRA6 at the cyst periphery and the organization of the cyst matrix. (A to J) Fluorescence intensity profiles of the cysts in Fig. 7A to J were generated to visualize the locations of GRA4 and GRA6 compared to DBA, which is a cyst wall marker on day 1 (A and B), day 2 (C and D), day 3 (E and F), day 7 (G and H), and day 10 (I and J). The dotted black lines define the cyst wall region as defined previously for each day. The dotted red line indicates the middle of the cyst wall, which corresponds to the peak DBA fluorescence intensity. Download FIG S7, TIF file, 1.6 MB. Copyright © 2019 Guevara et al. 2019 Guevara et al. This content is distributed under the terms of the Creative Commons Attribution 4.0 International license.

### A model of GRA localization dynamics during the formation of the cyst wall and cyst matrix.

**FIG 8:**
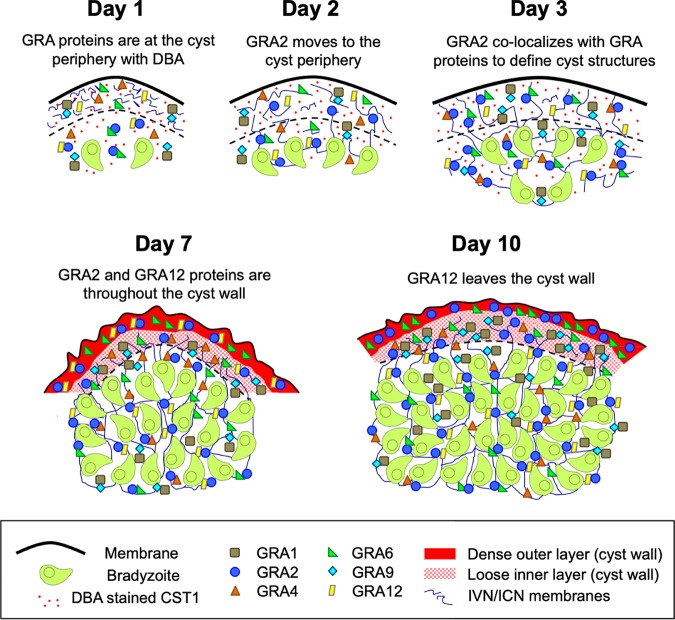
A model of GRA localization dynamics during the formation of the cyst wall. This model depicts the location(s) of GRAs during cyst development at 1 day, 2 days, 3 days, 7 days, and 10 days postdifferentiation. The patterns of colocalization of GRAs in the cyst wall and cyst matrix are shown by linked GRAs observed at different times of cyst development. At day 1, GRA proteins are at the cyst periphery with DBA, except for GRA2. At day 2, GRA2 begins to move toward the cyst periphery. At day 3, GRA2 colocalizes with GRA proteins to define the cyst matrix and cyst wall compartments. At day 7, GRA2, GRA6, and GRA12 were localized throughout the cyst wall, while GRA1, GRA4, GRA6, and GRA9 were localized only in the loose inner layer of the cyst wall. At day 10, GRA12 leaves the cyst wall and is found as puncta within the cyst matrix colocalized with GRA2.

A summary of the dynamic localization and colocalization of IVN-associated GRAs during cyst development and maturation is shown in a proposed model (Fig. 8). In 1-day cysts, IVN GRAs, except for GRA2, and DBA stain were concentrated at the cyst periphery. GRA9 and GRA1 were colocalized and were more concentrated at the cyst periphery than CST1-stained DBA. GRA2 transitioned to the cyst periphery by day 2, and the relative concentrations of GRA proteins at the cyst periphery were similar to 1-day cysts, though slightly reduced in magnitude, indicating that GRA proteins were beginning to accumulate in the cyst matrix by day 2. This pattern continued to day 3, which was the first time when the relative accumulation of the GRA proteins at the periphery fell below the CST1 DBA stain ratio (cyst periphery/cyst interior). Thus, prior to day 3, with the exception of GRA2, the IVN GRA proteins were primarily cyst wall components. Moreover, day 3 marked the first time when GRA2 was strongly colocalized in the cyst matrix and at the periphery with several GRAs (GRA4, GRA6, and GRA9), suggesting that GRA proteins in the developing cyst wall were concentrated by day 3 and were seeking their interacting partners to mature the cyst wall and to organize the cyst matrix. In contrast, GRA2 and GRA12 colocalized in prominent puncta in the cyst matrix at all times of cyst development but were colocalized in the cyst wall only on day 7. GRA2 localization and colocalization are likely to be important for the development of the cyst wall and the organization of the cyst matrix based on the defects we observe in Δ*gra2* cysts and the known role of GRA2 in tubulating the IVN membranes (40, 46). In mature cysts (7 days old or older), GRA2, GRA6, and GRA12 are localized throughout the cyst wall, while GRA1, GRA4, and GRA9 are concentrated in the loose inner layer of the cyst wall. GRA2 and GRA12 are found as puncta in the cyst matrix, while GRA1, GRA4, GRA6, and GRA9 are found continuously throughout the cyst matrix. In 10-day-old mature cysts, GRA12 dynamically leaves the cyst wall and is seen colocalized with GRA2 in cyst matrix puncta. Throughout cyst development, GRA1 colocalized with GRA9 in the cyst matrix and in the cyst wall, suggesting that the peripheral association of GRA1 with IVN membranes could be mediated through its association with GRA9.

## DISCUSSION

Environmental cues heavily influence the regulated programmed state of *Toxoplasma* that dictates the parasite’s life stage (54–58). The bradyzoite-stage cyst structure maintains chronic infection (59) and presents a therapeutic barrier for cyst clearance (60). Our study tracked the location(s) of IVN-associated GRA1, GRA2, GRA4, GRA6, GRA9, and GRA12 from differentiation of the PV to the mature 10-day-old *in vitro* cyst. Our findings confirm a recent proteomic study of the cyst wall that identified IVN-associated GRA2, GRA4, GRA9, and GRA12 in the cyst wall of *in vitro* cysts matured for 8 days (29). Notably, this proteomic study did not identify GRA1 or GRA6 in the cyst wall. However, GRA1 and GRA6 have been previously localized to the cyst wall via cryo-immunolabeling (19) or gold particle immunoelectron microscopy (2), respectively. The membrane tubules of the ICN present in the cyst matrix and in the cyst wall resemble tachyzoite-stage IVN membranes (18, 19), and the IVN membranes of the tachyzoite-stage PV have been previously speculated to play a role in cyst development (19, 47, 51). However, this question has not been previously investigated.

*Toxoplasma* forms a glycosylated cyst wall beneath a limiting cyst membrane. This compartmentalization is unusual for microbes that form walled structures. The majority of wall-building microbes utilize a limiting membrane as a platform to build the wall outside the limiting membrane (18, 61, 62). Cyst wall protein cargo could be delivered to the developing cyst wall in small or large vesicles that are secreted by bradyzoite-stage parasites (18, 19, 29). However, the precise mechanisms that deliver cyst wall cargo into the cyst wall compartment beneath the limiting cyst membrane still need to be determined.

GRA2 tubulates vesicles into highly curved membranes *in vitro* (46). Correspondingly, in the absence of GRA2 expression, IVN membranes are shaped into large membrane vesicles instead of the highly curved tubular IVN membrane structures (40). GRA6 was previously shown to relocalize, moving from the PV interior to concentrate beneath the PVM in tachyzoite-stage PVs that lacked expression of GRA2, suggesting that IVN membranes devoid of GRA2 compacted beneath the PVM (33). Our data revealed the absence of GRA2 at the cyst periphery and the presence of GRA4, GRA6, GRA9, and GRA12 at the cyst periphery prior to day 2 of cyst maturation. These results suggest that GRA2 was selectively removed from the IVN membranes after differentiation of the tachyzoite-stage PV, while GRA4, GRA6, GRA9, and GRA12 were retained in GRA2-devoid membranes that relocalized beneath the limiting membrane. We hypothesize that GRA2 exclusion from the IVN membranes after differentiation rapidly creates and defines the initial cyst wall compartment where cyst wall cargo will be delivered. This hypothesis is attractive because it localizes membranes beneath the limiting cyst membrane that could potentially support vesicular trafficking necessary to deliver cyst wall cargo secreted by bradyzoites in small and large vesicles (17, 19), and this hypothesis is consistent with our data showing that the initial cyst wall compartment was rapidly defined by 1 day postdifferentiation (five layers thick) as a distinct compartment that was nearly as thick (>80%) as the cyst wall compartment of 2- to 10-day-old *in vitro* cysts (six layers thick).

A previously proposed model for how the cyst wall could develop is based on a CST1 protein scaffold. CST1 is hypothesized to bind to the cyst matrix face of the limiting cyst membrane and act as a scaffolding protein for the construction of the cyst wall (51). CST1 possesses a mucin domain that is essential for the granular appearance of the cyst wall (17). The CST1 mucin domain is heavily glycosylated (28), and this explains why DBA selectively stains CST1 and the cyst wall (17). The glycosylated mucin domain of CST1 could provide extensive carbohydrate bonding interactions with other cyst wall-localized proteins (19). However, whether or how CST1 associates with the limiting cyst membrane early after differentiation is unknown. Our data suggest DBA stain, like CST1, is present throughout the cyst wall compartment early after differentiation and during cyst maturation. In addition, in mature *in vivo* cysts, CST1 is localized with granular material uniformly distributed throughout the wall (51). If CST1 has the capability to associate with membranes and build a protein-based scaffold from the membrane, then it is likely that CST1 would associate not only with the limiting cyst membrane but also with other membranes present in the developing cyst wall. The frequent appearance of membrane tubules, vesicles, and deep invaginations of the limiting cyst membrane in electron microscopy images of the mature cyst wall (18, 19, 29, 63–65) suggests that the cyst wall contains many membrane compartments. Our hypothesis of GRA2 exclusion as the driving force to create a new membrane system beneath the limiting cyst membrane after differentiation of the PV would provide bidirectional membrane scaffolds for cyst wall construction. This model is attractive because a CST1 scaffold would need to link the spaces between two membranes in the developing cyst wall, rather than span the full thickness of the cyst wall from the limiting cyst membrane to the cyst matrix.

GRA2 was localized to puncta in the cyst matrix, rather than in a continuous matrix pattern. Our data revealed a disruption of the organized structured matrix staining pattern in Δ*gra2* cysts that were matured for more than 2 days. These findings suggest that the matrix pattern may involve GRA2-positive puncta. Based on GRA2’s ability to tubulate membranes to establish the IVN in the tachyzoite-stage PV, GRA2’s relocalization to the cyst matrix early during cyst development could potentially enhance the mechanisms that tubulate the ICN membranes in the cyst matrix. Our results show that the transfer of GRA2 from the cyst matrix to the cyst wall coincided with the initial visualization of the GRA4, GRA6, and GRA9 matrix staining pattern between days 2 and 3 postdifferentiation. Our results suggest that the ICN may form initially in the cyst matrix before it is established in the cyst wall. While it is tempting to speculate that the matrix pattern in the cyst reflects the localization of the ICN, the relationship of the matrix pattern to the ICN membrane structures remains to be determined.

The intimate colocalization relationship of GRA12 and GRA2 to puncta in the cyst matrix suggests a critical role for GRA12 with GRA2 in organizing membrane structures in the cyst matrix. However, GRA12 was localized at the periphery earlier than GRA2 and was not well colocalized in the cyst wall with GRA2 until cysts had matured for 7 days, and then by 10 days, remarkably, GRA12 was not detected in the cyst wall. Interestingly, cysts matured *in vitro* after alkaline switch differentiation initially show limited oral infectivity after 5 days of development. This infectivity was significantly increased by day 7 postdifferentiation, and by day 9 of cyst development, *in vitro* cysts exhibited oral infectivity equivalent to or greater than oral infectivity of *in vivo* cysts (24). It is tempting to speculate that GRA12 colocalization with GRA2 in the cyst wall represents a maturation step of the cyst wall and that the relocalization of GRA12 from the cyst wall to the cyst matrix by day 10 represents a late step in cyst maturation.

The dynamic transition of GRA12 from the cyst wall into the cyst matrix raises intriguing possibilities as to the function of GRA12. In the tachyzoite-stage PV, GRA12 is required for resistance against host gamma interferon, and it was proposed to be essential for the acquisition of protein or lipid cargo from the host (47). In the tachyzoite-stage PV, GRA2 is required for sufficient ingestion of host cytosolic proteins, and it was proposed to have a role in trafficking host molecules across the PVM (39). However, at this time, it is unknown whether GRA2-dependent cyst mechanisms also ingest host cytosolic proteins. Our findings suggest that GRA2 and GRA12 intimately associate to establish key structures in the cyst matrix and participate in the development of the cyst wall.

To assess the proximity of IVN GRAs within the cyst wall, we developed a quantitative method to define the cyst wall region and measure the fluorescence intensity in individual cysts to locate IVN GRAs in relation to the DBA-stained cyst wall. The mature cyst wall is organized in two filamentous layers, an outer layer that lies beneath the limiting cyst membrane and is more densely compacted than the inner layer of the cyst wall that faces the cyst matrix (19). Using this mature cyst wall description in conjunction with our cyst wall analysis, GRA2, GRA6, and GRA12 were found in both cyst wall layers. In contrast, GRA1, GRA4, and GRA9 were found predominantly in the inner cyst wall layer that faces the cyst matrix. We also identified an intriguing colocalization relationship between GRA1 and GRA9 in both the cyst matrix and cyst wall. GRA1 association with GRA9 could potentially explain why the soluble GRA1 protein peripherally associates with the IVN membranes in the tachyzoite-stage PV (40), and possibly with the ICN membranes in the cyst. Notably, we observed a strong correlation between IVN-associated GRAs localizing to the inner cyst wall and those that were observed to form the matrix pattern in the cyst. The inner cyst wall contains membrane tubules similar to the ICN that are connected to the cyst matrix (18, 19). The transition of GRA2 from the cyst matrix to the cyst wall (between day 2 and day 3 postdifferentiation), or alternatively, the transition of GRA12 from the cyst wall to puncta in the cyst matrix (between day 7 and day 10 postdifferentiation) could mark the establishment or maturation of these tubule connections.

During the first 7 days of cyst development, the cyst periphery/cyst interior ratio of DBA staining of the major cyst wall protein CST1 was remarkably consistent, ranging between 1.02 and 1.26. However, between day 7 and day 10, the cyst periphery/cyst interior DBA fluorescent intensity ratio increased from 1.1 to 1.8. These results suggest the cyst wall compartment was being built at a similar rate during the first 7 days of cyst development, and then the wall matured between 7 and 10 days. The loss of GRA12 from the cyst wall between day 7 and day 10 suggests that GRA12 played a role in the development and maturation of the cyst wall and also that GRA12 is actually not a component of the mature cyst wall. GRA1, GRA4, GRA6, GRA9, and GRA12 on day 1 and day 2, and GRA2 on day 2, exhibited cyst periphery/cyst interior ratios that were greater than the ratio for the cyst wall protein CST1, indicating that these GRA proteins were components of the developing wall early during cyst development. This pattern reversed after day 3, as the cyst matrix developed, and by day 10, the GRA1, GRA2, GRA4, GRA6, GRA9, and GRA12 cyst periphery/cyst interior ratios were 0.28, 0.30, 0.46, 0.54, 0.42, and 0.13, respectively. The localization of IVN GRA1, GRA2, GRA4, GRA6, GRA9, and GRA12 to the cyst wall and cyst matrix during cyst development supports the hypothesis that the IVN develops into the cyst stage ICN, which is found in the cyst wall and cyst matrix (19). To confirm this hypothesis in future studies, it will be important to assess cyst development using superresolution microscopy and to assess GRA localization and colocalization using gold particle immunoelectron microscopy methods. The quantitative methods we developed to define the cyst wall region and localize and measure fluorescent intensity in each layer of the cyst structure provide another method to assess whether a cyst protein should be identified as a cyst wall protein, a cyst matrix protein, or a protein that is present in both the cyst wall and cyst matrix. Our results suggest that proteins that exhibit a cyst periphery/cyst interior ratio greater than 1.0 are likely to be cyst wall proteins, and proteins that exhibit a cyst periphery/cyst interior ratio less than 0.20 are likely to be cyst matrix proteins. Proteins with ratios between 0.20 and 1.0, and evidence of actual localization in wall and matrix layers, should be regarded as proteins that occupy both the cyst wall and cyst matrix compartments.

Previously identified cyst wall and/or cyst matrix proteins include ROP21 and ROP27 (66), GRA19 and GRA20 (67), MCP3 and CST2-CST6 (29), and MCP4 and BPK1 (68). Deletion of CST2 markedly reduced cyst burdens (29). Deletion of ROP27 reduced cyst burden (69), and ROP27 localized in puncta in the cyst matrix (66), similar to the localization of GRA2 and GRA12. Deletion of ASP5 or GRA12 reduced DBA staining intensity at the cyst periphery/wall (47, 70). In addition, genetic deletion of several *Toxoplasma* rhoptry (ROP) or IVN-associated GRA proteins was recently shown to markedly reduce cyst burdens (47, 50, 66, 69), Here, we show that deletion of GRA2, GRA4, GRA6, GRA9, and GRA12, but not GRA1, reduced DBA staining intensity at the cyst periphery relative to the cyst interior by day 3 postdifferentiation. The deletion of GRA2 also decreased the relative accumulation of GRA4 and GRA6 at the cyst periphery. These broad defects in the development of the cyst wall suggest that GRA proteins that strongly associate with the IVN (GRA2, GRA4, GRA6, GRA9, and GRA12) play globally important roles in mechanisms necessary for the development and maturation of the cyst wall.

## MATERIALS AND METHODS

### Parasite and cell culture.

Type II Prugniaud (Pru) background Toxoplasma gondii parasites were maintained *in vitro* by serial passage of tachyzoites in human foreskin fibroblast (HFF) monolayers (ATCC SCRS-1041.1) cultured in Eagle’s modified essential medium (EMEM) (Lonza) containing 1% fetal bovine serum (FBS) (Life Technologies), 2 mM glutamine, 100 U/ml penicillin, and 100 μg/ml streptomycin at 36°C in 95% air and 5% CO_2_. HFF cells were maintained in EMEM containing 10% FBS (HyClone), 2 mM glutamine, 100 U/ml penicillin, and 100 μg/ml streptomycin at 37°C in 95% air and 5% CO_2_. The parental Pru strain PruΔ*ku80* was previously made transgenic for green fluorescent protein (GFP) under the control of the LDH2 bradyzoite stage-specific promoter (53). Strains used in this study were developed using the Δ*ku80* knockout strain of the type II Pru strain using previously described methods (Table 1) (47, 50, 71).

**TABLE 1:** Parasite strains

Strain designation	Relevant genotype	Reference
PruΔku80	PruΔ*ku80*Δ*hxgprt*	50
PruΔgra1	PruΔ*ku80*Δ*gra1*::*HXGPRT*	This study
PruΔgra2	PruΔ*ku80*Δ*gra2*::*HXGPRT*	47
PruΔgra4	PruΔ*ku80*Δ*gra4*::*HXGPRT*	50
PruΔgra6	PruΔ*ku80*Δ*gra6*::*HXGPRT*	50
PruΔgra9	PruΔ*ku80*Δ*gra9*::*HXGPRT*	47
PruΔgra12	PruΔ*ku80*Δ*gra12*::*HXGPRT*	47
PruΔgra12/GRA12_II_	PruΔ*ku80*Δ*gra12::HXGPRT*::*GRA12_II_^FLHA^*	This study

### Deletion of GRA1.

Targeted deletion of GRA1 (Table 1) was developed using the PruΔ*ku80* strain as previously described (see Table S1 in the supplemental material) (47, 50, 71). Briefly, GRA1 gene locus knockout targeting plasmid was assembled in yeast shuttle vectors pRS416 using yeast recombinational cloning to fuse in order three distinct PCR products with 33-bp crossovers; a 5′ *GRA1* target gene flank, the *HXGPRT* selectable marker, and a 3′ *GRA1* target flank (Fig. S1A) (72). Knockout plasmids were engineered to delete at least 200 nucleotides of the 5′ untranslated region (UTR) and the complete coding region of the *GRA1* gene locus as defined in the ToxoDB.org database (52). All oligonucleotide primers used to construct knockout targeting plasmids and the ToxoDB nucleotide definition of GRA1 gene locus deletion are listed in Table S1. Targeting plasmids were validated by DNA sequencing, and the plasmids were linearized at restriction sites inserted at the 5′ end of the 5′ targeting flank (Fig. S1A). Linearized targeting plasmids were transfected by electroporation into tachyzoites of the PruΔ*ku80* strain. *GRA1* knockouts were selected in 50 μg/ml mycophenolic acid and 50 μg/ml xanthine. Drug-selected strains were cloned by limiting dilution 30 days after transfection. *GRA1* knockouts were validated by genotype analysis using a PCR strategy (shown in Fig. S1A) to measure the following: (i) in PCR 1, targeted deletion of the coding region of the targeted gene (GRA1PDF and GRA1PDR primers); (ii) in PCR 2, correct targeted 5′ integration (GRA1PCXF and 5′DHFRCXR primers); and (iii) in PCR 3, correct targeted 3′ integration (3′DHFRCXF and GRA1PCXR primers) using knockout validation primers shown in Table S1.

10.1128/mSphere.00487-19.8 TABLE S1 DNA primers for construction of gene replacement plasmids. Download Table S1, DOCX file, 0.03 MB. Copyright © 2019 Guevara et al. 2019 Guevara et al. This content is distributed under the terms of the Creative Commons Attribution 4.0 International license.

### Complementation of Δ*gra12*.

Complementation plasmids were designed to complement Δ*gra12* through targeted chromosomal integration and expression of wild-type GRA12 at the endogenous locus using previously described methods (Fig. S1D) (50, 69). Complementation plasmids were developed in the pRS416 yeast shuttle vectors using yeast recombination to fuse, in order, a 5′ UTR target flank, the complementing gene of interest, and the 3′ UTR target flank (Fig. S1D). Oligonucleotide DNA primers (Table S1) were used to generate the complementing genes, synthesized as one PCR product. Following plasmid assembly by yeast recombinational cloning, targeting plasmids were validated by DNA sequencing. Prior to transfection, plasmids were linearized via the unique restriction site at the 5′ end. Parasites were cultured for 2 days in normal infection medium, and cultures were then switched to selection medium containing 30 mg/ml of 6-thioxanthine (6TX) and cloned 30 days after transfection by limiting dilution. Accurate targeting of complementing genes into the endogenous locus was validated by genotype analysis using PCR assays (strategy shown in Fig. S1D) to measure the following: (i) in PCR 4, correctly targeted 5′ integration (GRA12PCXF2 and UGR12XR primers) and (ii) in PCR 5, correctly targeted 3′ integration of the complementing gene at the endogenous locus (HASEQF and GRA12CXR primers) using oligonucleotide DNA validation primers (Table S1).

### Immunofluorescence assay.

For visualization of dense granule proteins within the tachyzoite-stage PV, HFF cells were cultured on circular micro cover glass (Electron Microscopy Sciences) and infected with parasites for 24 h. Samples were fixed in 4% paraformaldehyde for 10 min, permeabilized with 0.01% saponin (Sigma) for 10 min, and blocked with 10% FBS for 20 min. All samples were incubated with 1:500 dilution of primary mouse monoclonal anti-GRA1 or 1:50 dilution of anti-MAG1 antibody. Preparations were washed three times with Dulbecco’s phosphate-buffered saline (DPBS) supplemented with Ca^2+^ and Mg^2+^ (HyClone) and incubated for 1 h at room temperature (RT) with a 1:1,000 dilution of secondary goat anti-mouse IgG antibodies conjugated to Alexa Fluor 594. All samples were mounted in Slowfade Gold antifade with DAPI (4′,6′-diamidino-2-phenylindole) (Life Technologies) and imaged at 100× with a Nikon A1R SI confocal microscope (Nikon, Inc.). PVs were located using differential interference contrast (DIC) microscopy. Confocal images as raw .nd2 files were imported and minimally processed for brightness in Fiji (73).

### *In vitro* cyst differentiation assay.

Tachyzoites were differentiated *in vitro* into bradyzoites within cysts essentially as previously and elegantly described by Tobin and colleagues (22). Differentiation medium contained Roswell Park Memorial Institute medium (RPMI) without bicarbonate supplemented with 2.05 mM l-glutamine (HyClone), 20 mM HEPES (free acid) (IBI Scientific), 1% XL-glutamine (a long-lasting stable form of glutamine) (VWR), 1% FBS, and 1% penicillin-streptomycin. The pH of the differentiation medium was adjusted to 8.1 with sodium hydroxide and filter sterilized. HFF cells were cultured on circular micro cover glass until confluent (Electron Microscopy Sciences), and confluent monolayers were infected with type II Pru parasites at a multiplicity of infection (MOI) of ∼0.5. Three hours after infection, infected cells were washed once in DPBS supplemented with Ca^2+^ and Mg^2+^ and incubated in differentiation medium for 6 h, 1 day, 2 days, 3 days, 7 days, or 10 days at 37°C in ambient air. The medium was replaced with fresh medium on day 3 and day 7.

### Cyst immunofluorescence assay and cyst locating.

Infected cells were fixed in 4% paraformaldehyde for 10 min, and excess was quenched with 0.1 M glycine. Infected cells were permeabilized and blocked in 3% FBS−0.2% Triton X-100 for 30 min and continued throughout the experiment. All samples were incubated with a 1:500 dilution of primary rabbit monoclonal anti-HA-tagged antibody (Cell Signaling) to stain GRA12, or 1:1,000 dilution of primary mouse monoclonal anti-GRA1, anti-GRA2 antibody (74), or 1:1,000 dilution of primary rabbit anti-GRA4 (41), anti-GRA6 (41), anti-GRA9 (43) (antibodies purchased from Biotem, Apprieu, France, or kindly provided by L. D. Sibley, Washington University School of Medicine, St. Louis, MO, or W. Daübener, Heinrich Heine Universität, Düsseldorf, Germany). Preparations were washed three times with DPBS supplemented with Ca^2+^ and Mg^2+^ and incubated for 1 h at RT with a 1:1,000 dilution of secondary goat anti-rabbit (H+L) (Thermofisher) and goat anti-mouse IgG (H+L) antibodies conjugated to Alexa Fluor 647 (Cell Signaling). All samples were incubated with a 1:250 dilution of rhodamine-labeled Dolichos biflorus agglutinin (Vector Laboratories) for 1 h at RT. Samples were mounted in Slowfade Gold antifade with DAPI (Life Technologies) and then imaged with a Nikon A1R SI confocal microscope (Nikon, Inc.) using an Apo TIRF 100× Oil DIC N20 objective. Cysts were randomly selected for analysis by locating cysts using DIC microscopy. Bradyzoite differentiation in cysts was confirmed by GFP^+^ bradyzoites. The focal plane (from a Z-stack) selected for quantification was from the middle of the cyst, where the cyst size is maximal (Movie S1). Raw .nd2 files of cyst images were imported into Fiji for processing. Images were minimally processed for brightness (image → adjust → color balance) in Fiji (73). The number of cysts for each strain analyzed in each experiment is shown in the figure legends.

10.1128/mSphere.00487-19.10 MOVIE S1 Movie of a 7-day cyst stained with DBA and GRA6 (Z-stack). Download Movie S1, MOV file, 0.04 MB. Copyright © 2019 Guevara et al. 2019 Guevara et al. This content is distributed under the terms of the Creative Commons Attribution 4.0 International license.

### Quantification of cysts positive for GRA colocalization.

Each imaged cyst was analyzed for spots of yellow, which indicated colocalization between GRA2 and GRA4/GRA6/GRA9/GRA12 or GRA9 and GRA1. The numbers of cysts that were positive for yellow spots were counted out of the total number of cysts (*n *= 10 to 34 cysts) and multiplied by 100 to be presented as a percentage of cysts that showed colocalization.

### Cyst fluorescence intensity profiles.

Raw .nd2 image files were imported into Fiji to measure fluorescence intensity parallel to the cyst wall using a new Fiji macro (Text S1). To read the macro, select the IJ1 Macro language under the drop-down language menu. Images were cropped to isolate each cyst. The macro was written to generate a reliable mask of the cyst, adjacent to (just slightly outside) the cyst wall using the DBA-rhodamine channel (Fig. S2A and Fig. S2C, panel 1). The DBA-rhodamine channel was used to threshold the cyst, and holes were filled inside to obtain a continuous mask of the whole cyst. Successive layers, denoted by the letter L, were generated based on the original mask, growing or shrinking by dilating or eroding morphological operations. The mask was duplicated by dilation to create the first region between two layers (L-1 and L0), which was approximately one pixel thick (Fig. S2B). The following parameters were selected to be measured: feret diameter and mean fluorescence intensity. The fluorescence intensity of each region was measured for a selected fluorescent channel: DBA, GFP, GRA1, GRA2, GRA4, GRA6, GRA9, or GRA12. The macro-generated layers, approximately one pixel thick, within the cyst until the minimum area of the (shrinking) layer reached 1,000 pixels^2^. Fifteen layers were created by dilation to measure the fluorescence intensity outside the cyst, which provided the background fluorescence intensity (Fig. S2C). All data were imported into Excel for analysis. The following calculations were done. (i) All background layers had similar levels of fluorescence intensity, but for consistency, the fluorescence intensity of the region between L-15 and L-14 was used as the background level, which was subtracted from all other fluorescence intensity values. (ii) Next, we calculated the percentage of maximum fluorescence intensity by dividing all of the fluorescence intensity values by the highest fluorescence intensity and multiplying by 100. (iii) Distance from the cyst wall was determined by the feret diameter, which measured the distance between two parallel places that restricted the cyst perpendicular to that direction. This allowed for cysts to be measured in the same way regardless of how they were shaped or positioned. The feret distance (diameter) from layer -15 was subtracted from layer -1 (which was determined as the total cyst, see the explanation below in “Cyst wall definition and analysis”) and divided by two (radius) to determine the shift from zero. (iv) Next, we calculated the position of each layer (noted as layer X) using this formula: ((layer -15 feret diameter) – (layer X feret diameter))/2 + shift from zero value. The calculated percentages of maximum fluorescence intensity and distance (in micrometers) values were imported and graphed in Prism.

### Cyst wall definition and analysis.

First, we developed parameters to identify and define the region of the cyst wall. The position of the outer region of the cyst wall was identified by DBA fluorescence, which identified the CST1 wall protein target of DBA staining, while the position of the inner wall region was identified by GFP fluorescence, which identified the bradyzoites that were inside the cyst wall. We quantitatively defined the cyst wall region outer and inner boundary determined by the first layer with less than 50% of maximum fluorescence intensity of DBA (outermost layer of the cyst wall) or GFP (innermost layer of the cyst wall), respectively. The rationale for selecting these parameters is discussed in the Results section. This standardized analysis was performed for each cyst assessed for each time point on or after day 1. One-day cysts revealed five layers (L-1, L0, L1, L2, and L3) that defined the cyst wall region, while 2-day-, 3-day-, 7-day-, and 10-day cysts revealed six layers (L-1, L0, L1, L2, L3, and L4) that defined the cyst wall region, denoted by dotted vertical black lines in the graphs in the figures. Consistently, the peak of DBA fluorescence was observed in layer L1 in 1-day cysts (third symbol from zero on the graphs, i.e., Fig. 2E) or in layers L1 and L2 for 2-day, 3-day, 7-day, and 10-day cysts (third and fourth symbols from zero on the graphs, i.e., Fig. 6E), and the peak of DBA fluorescence is marked by a dotted vertical red line in the graphs in the figures. Next, we evaluated the locations of proteins in comparison to one another based on fluorescence intensity measured at the same time in the cyst. This cyst wall analysis was used to determine whether two proteins were observed in the same layer of the cyst wall, approximately 1 pixel thick.

### Cyst total fluorescent intensity quantification assay.

Raw .nd2 image files were imported into Fiji to measure total fluorescence intensity at the cyst periphery and within the cyst interior (Fig. S3) using a new Fiji macro (Text S1). Images were cropped to isolate each cyst. The macro was written to generate a reliable mask of the cyst, slightly outside the cyst wall using the DBA-rhodamine channel (Fig. S3A). The DBA-rhodamine channel was used to threshold the cyst, and holes were filled inside to get a continuous mask of the whole cyst. Successive layers were generated based on the original mask, growing or shrinking by dilating or eroding morphological operations. The following parameters were selected to be measured: area and mean fluorescence intensity. Based on the previous defined outer region, selection of layer L-1 was used to measure the fluorescence of the entire cyst (Fig. S3B). The cyst periphery was defined to be the cyst wall plus the two layers adjacent to the inner cyst wall, corresponding on day 1 to layers L-1 to L3 plus L4-L5 and on day 2 through day 10 to layers L-1 to L4 plus L5-L6 (Fig. S3B). The two layers closest to the cyst wall were added to include wall protein cargo near the cyst wall but not yet incorporated into the cyst wall. Fluorescence signals for DBA, GFP, GRA1, GRA2, GRA4, GRA6, GRA9, and GRA12 were measured. To measure background fluorescence, a circle was drawn using the freehand selection tool, and fluorescence was measured outside the cyst on three different sides. All data were imported into Excel for analysis. The following calculations were done. (i) To calculate the average intensity, the area of the measured region was multiplied by total fluorescence. (ii) To calculate the fluorescence background, the three average intensity measurements were averaged and multiplied by the area of the region measured. (iii) The fluorescence background was subtracted from the average intensity value. (iv) To determine the fluorescence at the cyst periphery (cyst wall plus two layers), the inside average fluorescence intensity (L5 for 1-day cysts or L6 for 2-day, 3-day, 7-day, and 10-day cysts) was subtracted from the entire cyst average fluorescence intensity (L-1). (v) Next, we calculated the ratio of fluorescence intensity by dividing the fluorescence intensity at the cyst periphery by the fluorescence intensity inside the cyst, termed the cyst interior. All ratios were entered and graphed in Prism. A ratio of <1 indicates that there is greater GRA fluorescence intensity in the cyst interior than at the cyst periphery, a ratio of 1 represents an equal fluorescence intensity at the cyst periphery compared to the cyst interior, and a ratio of >1 indicates there is greater fluorescence intensity at the cyst periphery than in the cyst interior. *P* values were calculated with a Student’s *t* test and indicated as follows: *, *P* < 0.05; **, *P* < 0.01; ***, *P* < 0.005; ****, *P* < 0.0001; NS, not significant.

### Cyst area analysis.

The surface area of each cyst was measured during quantification of total fluorescence intensity. Area values were measured at L-1 in differentiated 3-, 7-, and 10-day *in vitro* cysts. Cyst areas were measured in square micrometers and graphed in Prism (*n *= 24 cysts). *P* values were calculated with a Student’s *t* test.

### Statistical analysis.

Unpaired *t* tests were used to calculate *P* values. All calculations of averages, standard errors of the means (SEM), and *P* values were performed using GraphPad PRISM software version 5.0c.
