# Supplementary figures and images for: Toxoplasma gondii Intravacuolar-Network-Associated Dense Granule Proteins Regulate Maturation of the Cyst Matrix and Cyst Wall
Source: mSphere. 2019 Oct 16;4(5):e00487-19. doi: 10.1128/mSphere.00487-19 (PMC6796980; doi:10.1128/mSphere.00487-19)

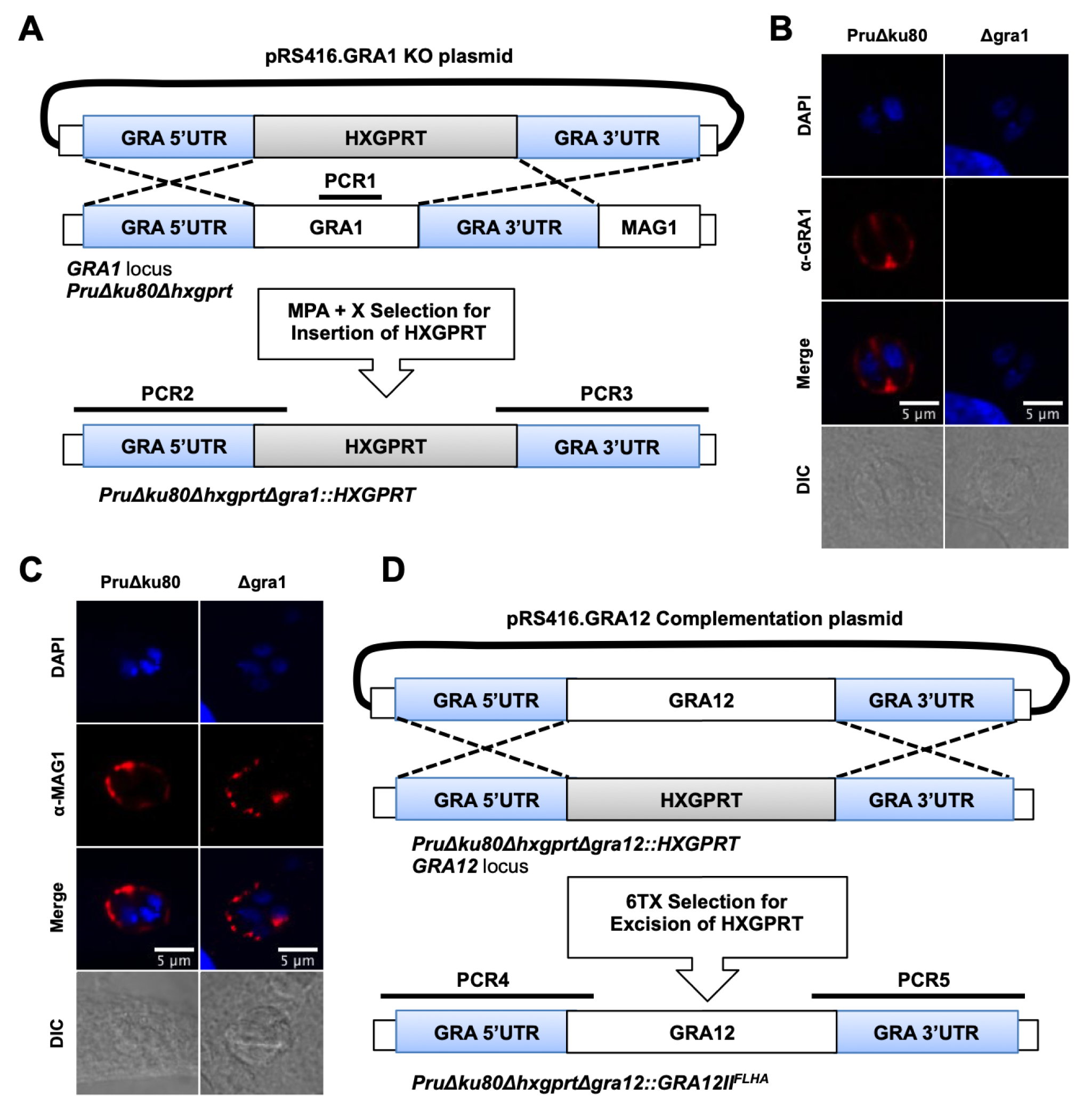

Supplement: FIG S1 [file mSphere.00487-19-sf001.tif]

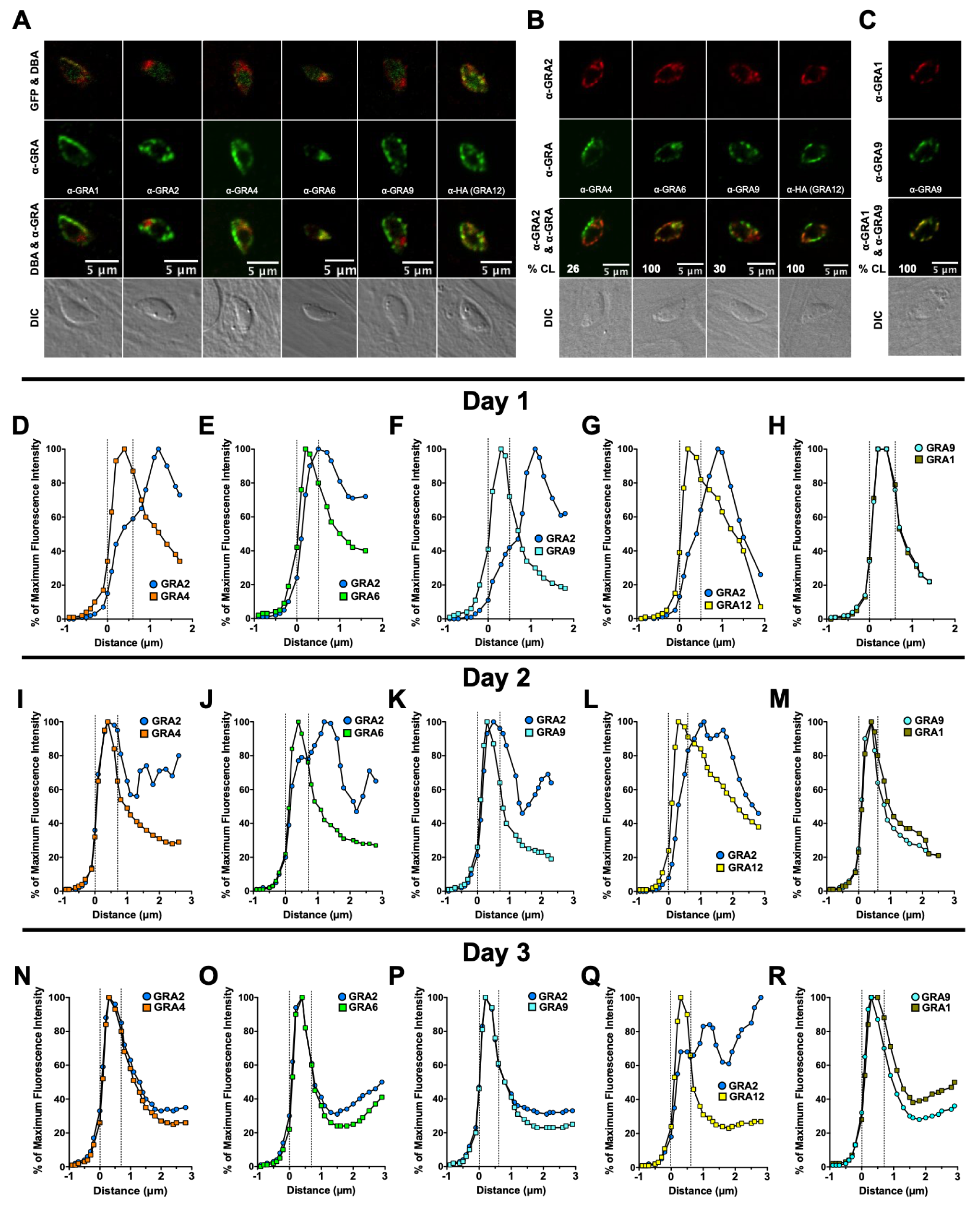

Supplement: FIG S4 [file mSphere.00487-19-sf004.tif]

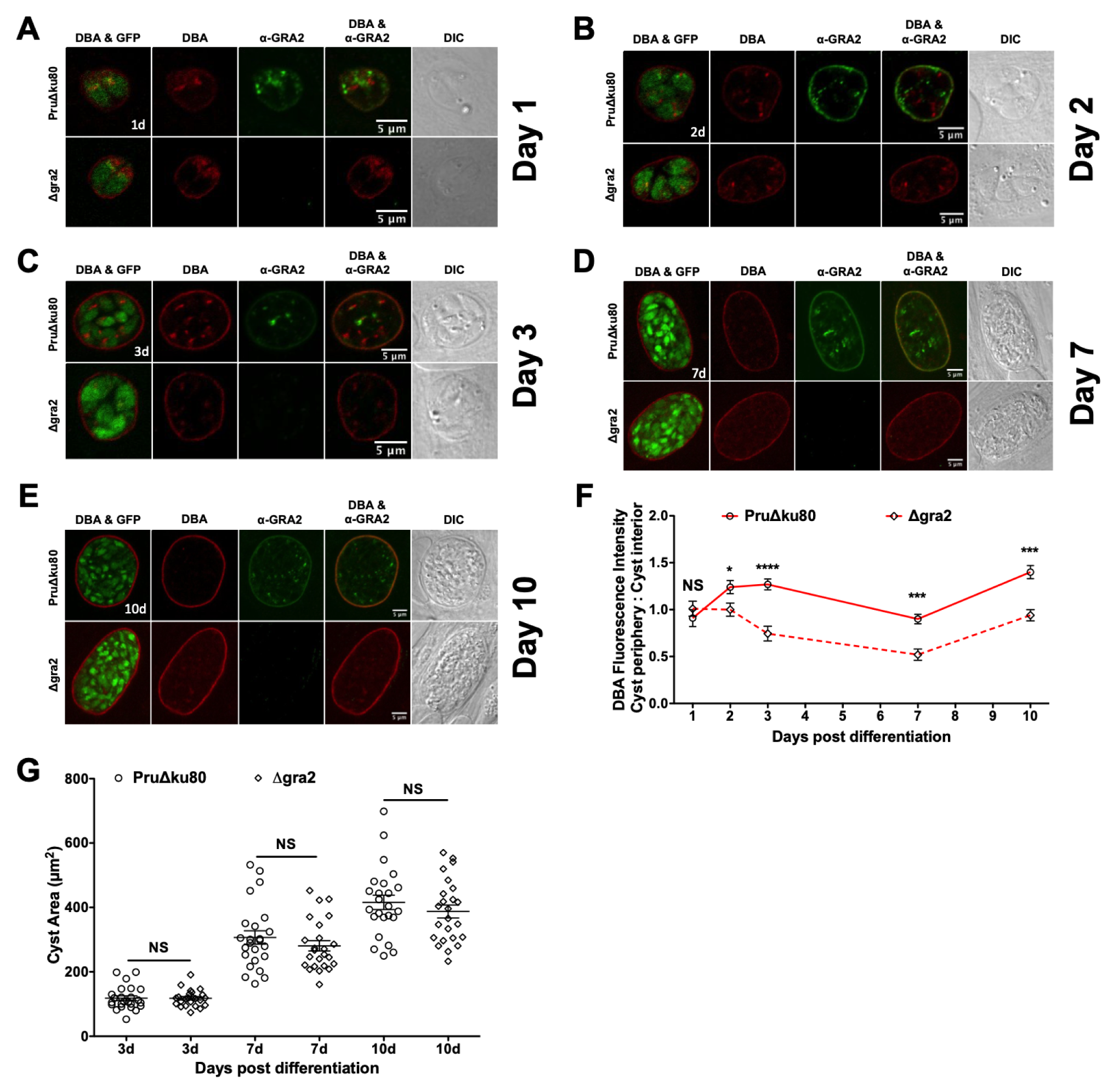

Supplement: FIG S6 [file mSphere.00487-19-sf006.tif]

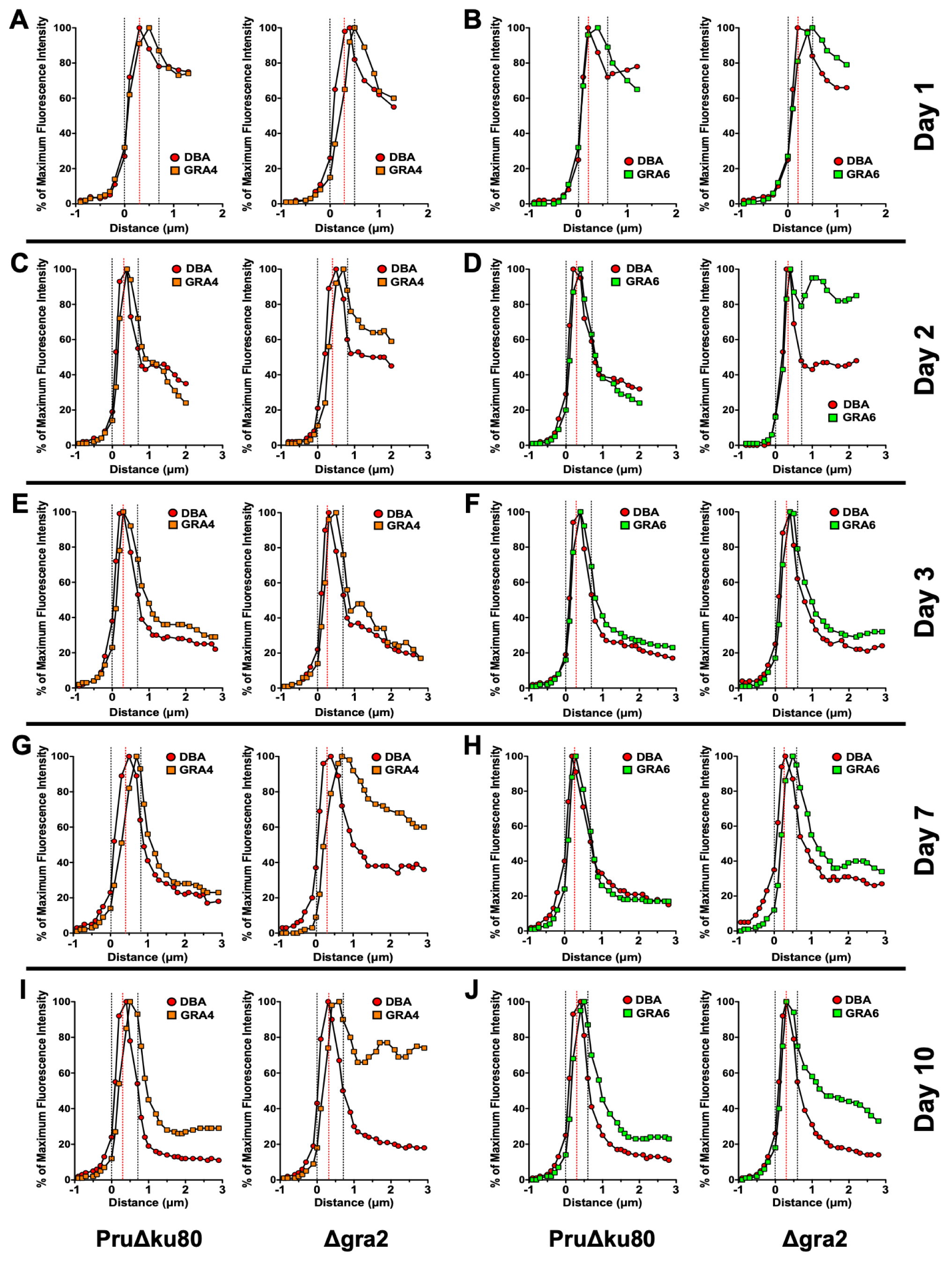

Supplement: FIG S7 [file mSphere.00487-19-sf007.tif]
