# Supplementary material for: Toxoplasma gondii Intravacuolar-Network-Associated Dense Granule Proteins Regulate Maturation of the Cyst Matrix and Cyst Wall
Source: mSphere. 2019 Oct 16;4(5):e00487-19. doi: 10.1128/mSphere.00487-19 (PMC6796980; doi:10.1128/mSphere.00487-19)
Supplement: TABLE S1 [file mSphere.00487-19-st001.docx]

**Table S1. DNA primers for construction of gene replacement plasmids.**

**Primers used to construct and validate knockout of GRA1.**

**Primer Sequence Primer Use Construct Corresponding locus KO**

**PMiniHXF** GATAAGCTTGATCAGCACGAAACCTTG HXGPRT cassette FP HXGPRT mini cassette NA

**PMiniHXR** CCGCTCTAGAACTAGTGGATCCC HXGPRT cassette RP

**GRA1PF1** *TTGGGTAACGCCAGGGTTTTCCCAGTCACGACG*GTTTAAAC**GGGTCGATGCTGAATCACTCGG** Pru Gra1 KO 5’ FP pRS416.pGRA1P TGME49_270250 chrVIII 5309421 to 5311590 (-)

**GRA1PR1** *GCGGGTTTGAATGCAAGGTTTCGTGCTGATCAA*ACTAGT**GAGCCTGCAAGACACTGGTTGG** Pru Gra1 KO 5’ RP

**GRA1PF2** *TTCTGGCAGGCTACAGTGACACCGCGGTGGAGG*ACTAGT**GGCGTACGGTAGTTGAAGTACCG** Pru Gra1 KO 3’ FP

**GRA1PR2** *GTGAGCGGATAACAATTTCACACAGGAAACAGC*GCGGCCGC**GCCGTTCCTATCATGTGTCAGCG** Pru Gra1 KO 3’ RP

**Primers used for validation of genotype**

**5’DHFRCXR**  **ACTGCGAACAGCAGCAAGATCG** 5' integration validation RP for all KO's

**3’DHFRCXF** **GTTGGCCTACGTGACTTGCTGATG** 3' integration validation FP for all KO's

**GRA1PCXF** **CTTCACAGTCCGGCACCACC** 5' integration validation FP

**GRA1PDF** **GGGGAACACGTATCGTGTGGAG** deletion validation FP

**GRA1PDR GCAGGTGAAGTAACATGGGGTACG** deletion validation RP

**GRA1PCXR ACCCTCCAGTGCACACGGTC** 3' integration validation RP

*Italicised nucleotides indicate regions of crossover in yeast recombination cloning, underlined nucleotides indicate restriction enzyme sites, and bold nucleotides indicate GRA1 specific priming targets (ToxoDB, version 27.0). FP and RP denote forward and reverse primers respectively.

**Primers used to construct plasmid for complementation and validation of complemented GRA12 mutant strain.**

**Primer Sequence Primer Use Construct Corresponding locus**

**Primers used for validation of genotype**

**GRA12PCXF2 CTTCTGGTTCGGGCCAGCAC** Pru 5' integration validation FP pRS416.UGRA12_II_^FLHA^ ME49_288650 chrlX 2,657,228 to 2,659,728 (-)

**UGR12XR**  **CCCTCATCGTGTAGCAGATTGCG** Pru 5' integration validation RP

**HASEQF ACCCATACGATGTTCCAGATTACGC** Pru 3' integration validation FP

**GRA12CXR AGAGTTGAGTGCCTACGTCCCTC** Pru 3' integration validation RP

* Bold nucleotides indicate priming regions specific to *Toxoplasma gondii* (ToxoDB, version 27.0). FP and RP denote forward and reverse primers respectively.
